# Mechanical memory operations in piezotransistive GaN microcantilevers using Au nanoparticle-enhanced photoacoustic excitation

**DOI:** 10.1038/s41378-021-00330-6

**Published:** 2022-01-24

**Authors:** Ferhat Bayram, Durga Gajula, Digangana Khan, Goutam Koley

**Affiliations:** 1 Holcombe Department of Electrical and Computer Engineering, Clemson University, Clemson, SC 29634 USA; 2 School of Electrical and Computer Engineering, Georgia Institute of Technology, Atlanta, GA 30332 USA

**Keywords:** Engineering, Electronic devices

## Abstract

Nonlinear oscillations in micro- and nanoelectromechanical systems have emerged as an exciting research area in recent years due to their promise in realizing low-power, scalable, and reconfigurable mechanical memory and logic devices. Here, we report ultralow-power mechanical memory operations utilizing the nonlinear oscillation regime of GaN microcantilevers with embedded piezotransistive AlGaN/GaN heterostructure field effect transistors as highly sensitive deflection transducers. Switching between the high and low oscillatory states of the nonlinear oscillation regime was demonstrated using a novel phase-controlled opto-mechanical excitation setup, utilizing a piezo actuator and a pulsed laser as the primary and secondary excitation sources, respectively. Laser-based photoacoustic excitation was amplified through plasmonic absorption in Au nanoparticles deposited on a transistor. Thus, the minimum switching energy required for reliable memory operations was reduced to less than a picojoule (pJ), which translates to one of the lowest ever reported, when normalized for mass.

## Introduction

The concept of mechanical computers has always fascinated researchers throughout the evolution of computing, especially attracting research interest since the demonstration of the first difference engine by Charles Babbage almost two centuries ago^1^. Over the years, the development of efficient and sufficiently miniaturized mechanical computing engines, a mechanical computer with logic and memory operations capabilities, has remained a challenging task for the research community, while silicon microelectronics-based memory cells and logic devices have continued to experience a dramatic reduction in size and power consumption, resulting in their widespread application in diverse fields^2^. With traditional silicon-based transistor technologies approaching their fundamental size and power dissipation limits in recent years, mechanical memory and logic devices, as alternatives to conventional computing, have once again started to garner significant research interest^3^. The capability of operating in harsh environments and the possibility of versatile logic operations in the same device are some advantages of mechanical memory and logic devices^4,5^. Considering the vast advancement in fabrication and instrumentation in recent years, memory and logic devices based on micro/nanoelectromechanical systems (MEMS/NEMS) and resonators may compete against current electronic transistor-based computing systems in terms of size, speed, and power consumption^2,4,6–8^.

The demonstrated memory and logic operations utilizing MEMS and NEMS are mainly based on their dynamic nonlinear behaviors originating from internal and external factors, such as large deformations and rotations^9^, boundary conditions^10^, nonlinear damping^11^, and parametric excitations^12^, which result in the operation of the oscillator in the nonlinear regime and creation of two stable states – one oscillatory and the other nonoscillatory, which facilitates memory operations. The transition between these stable states is achievable through various procedures, including changing the frequency or amplitude of the external excitations. Nonlinearity-based mechanical memory and logic operations have been investigated utilizing a single excitation source, including electrostatic^13–19^, electrothermal^5,20^, magnetomotive^6^, piezoelectric^7,21^, magnetic^22,23^, and piezo actuator-based actuations^24,25^. Regardless of the excitation source, they are all grouped as mechanical memory due to the mechanical oscillatory nature of the memory element. Most of these excitation techniques require either complex microfabrication processes and/or additional components to provide sufficient excitation, forcing the system to oscillate in the nonlinear regime^26^. In addition, changing the current status of multiple resonators is not feasible and efficient with a single external resonating source. To selectively operate an array of resonators for memory and logic operations, two external actuation sources can be utilized, where the resonators can be sufficiently excited to oscillate in the bistable region using one (primary) external source, while the other (secondary) can be used to switch between stable states acting as a “set and reset operator for a given resonator” using very little power. Optical excitation methodologies using the photoacoustic effect offer a noncontact, simple actuation technique; therefore, they are highly suitable for efficient dynamic mechanical memory applications.

Light-driven resonators have become a promising platform for developing photoacoustic spectroscopy applications^27–31^, offering several advantages, including noncontact actuation, easy integration, and zero background^32^. Photoacoustic excitation relies on the generation of acoustic waves through periodic expansion and contraction of photon absorbing media due to localized heating caused by exposure to a pulsed light source. The generated acoustic wave can be used to excite a mechanical oscillator very efficiently if the pulsing frequency of the light source matches the resonance frequency of the oscillator. Despite its several advantages compared to traditional excitation sources, the photoacoustic excitation technique has not been utilized to investigate the intrinsic nonlinearities of MEMS devices or their bistable operation.

Here, we report, for the first time, the investigation of dynamic mechanical memory operations utilizing ultrasensitive AlGaN/GaN heterojunction field effect transistor (HFET)-embedded GaN microcantilevers driven with single and multiple external oscillation sources, including primary excitation with a piezo actuator and secondary photoacoustic actuation with a pulsed laser. An AlGaN/GaN HFET embedded at the cantilever base was utilized to transduce microcantilever tip deflections into electrical signals; hence, it is called a “piezotransistive” microcantilever. In the mode of a single excitation source, the transition between the high and low stable states of the GaN microcantilevers in their intrinsic nonlinear regime was successfully demonstrated using either modulation of the piezo actuator bias, the laser beam location, or the laser power. In addition to single excitation-based switching, we experimentally demonstrated for the first time that two different excitation sources could be combined utilizing constructive and destructive interference properties to switch between the two stable states of the cantilever. While using the piezo actuator as the primary excitation source to oscillate the microcantilever in the bistable region, a pulsed laser-based secondary (photoacoustic) excitation was used to perform switching between the states. Moreover, the laser power to make the transition between states was reduced to ultralow levels following the deposition of Au nanoparticles, which strongly augmented photoacoustic signal generation due to plasmonic absorption.

Previously, we have shown that the cantilever dimensions have significantly affected the nonlinear cubic constant^9,33^, which determines the minimum excitation required to push an oscillator to the nonlinear regime^34^. Our results indicated that GaN microcantilevers with larger widths (>70 μm) at a fixed length of 250 μm exhibit strong softening-type nonlinearities where the resonance peak shifted to lower frequencies. On the other hand, hardening-type nonlinearities, where the resonance peak moves to higher frequencies, were dominant for cantilevers with smaller widths (<70 μm). Considering those earlier observations, two GaN microcantilevers, one with softening and one with hardening-type intrinsic nonlinearities in the first flexural mode, were selected to demonstrate mechanical memory operations in the present study.

## Results

### Device characterization

**Fig. 1:**
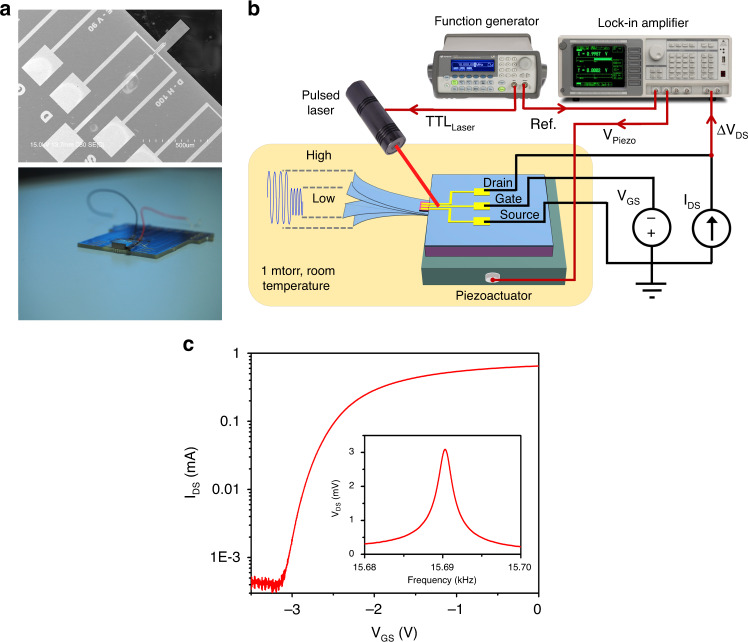
Experimental setup and characterization of GaN microcantilevers. **a** SEM image of the microcantilever (top) and photo of the piezo actuator attached underneath the microcantilever chip (bottom). **b** Schematic diagram of the experimental setup showing simultaneous piezo chip and photoacoustic excitation (using a pulsed laser) to switch cantilever states. **c** Electrical (I_DS_−V_GS_) and mechanical (resonance, inset) characteristics of a microcantilever with dimensions of 250 × 100 µm. The resonance frequency and the quality factor of the resonator are determined to be 15.690 kHz and 9600, respectively

**Fig. 2:**
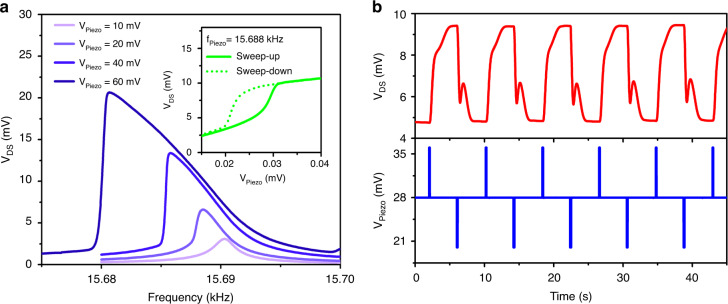
Memory operations using a piezo actuator. **a** Softening-type nonlinear resonance characteristics of the microcantilever under different piezo actuator biases. The inset shows the hysteresis behavior at a fixed frequency of 15.688 kHz. **b** Switching the cantilever’s stable states using the piezo actuator. A piezo bias of 28 mV was used to keep the cantilever in the bistable region. A bias differential of ±8 mV was added to the signal for 100 ms to switch the cantilever from one bistable oscillatory state to another. Due to intrinsic softening properties, all resonance curves in (**a**) were swept from high to low frequencies (sweep-down)

Figure 1a and Fig. S1a show scanning electron microscope (SEM) images of fabricated microcantilevers with dimensions (length × width) of 250 × 100 μm and 150 × 50 μm, respectively. The microcantilevers were tested using the generic experimental setup illustrated in Fig. 1b, where the microcantilevers were attached to a linear piezo actuator with dimensions of 5 × 5 × 2 mm. For our studies, the microcantilevers were placed inside a home-built vacuum chamber setup, as shown in Fig. S2, at a pressure of ~1 mTorr. Figure 1c and Fig. S1b display the I-V (current-voltage) characteristics of the AlGaN/GaN HFETs embedded at the bases of the aforementioned microcantilevers. When the microcantilevers were excited into oscillations (i.e., using a piezo actuator), alterations in the HFET drain-source voltage (V_DS_) caused by piezo-induced modulations of two-dimensional electron gas (2DEG) located at the AlGaN/GaN interface were measured using a lock-in amplifier^35–37^. Detailed investigations and analysis of the deflection transduction mechanism in AlGaN/GaN HFET-embedded GaN microcantilevers can be found elsewhere^35–39^. A schematic of the experimental setup used for piezo actuator-based excitation is shown in Fig. S3. The resonance characteristics of the microcantilevers in the linear regime were measured by applying a 4 mV ac (rms) bias to the piezo actuator, as shown in the inset of Fig. 1c and Fig. S1b. Increasing the drive amplitudes forced the microcantilevers to enter their intrinsic nonlinear territories. Figure 2a displays the resonance curves of the microcantilever with dimensions 250 × 100 μm at voltages (V_Piezo_) of 10, 20, 40, and 60 mV (rms) applied to the piezo actuator, indicating that softening-type nonlinearities are dominant at higher drive strengths, observable for 20 mV bias and beyond. On the other hand, hardening nonlinearity was exhibited from the microcantilever with dimensions of 150 × 50 μm at piezo actuator biases of 200 and 300 mV, as shown in Fig. S3a.

### Memory operations utilizing piezo actuator-based excitations

Two distinct features of Duffing-type nonlinearities, bifurcations and hysteresis, have been observed with GaN microcantilevers in the frequency domain^9,33,34^. In the nonlinear regime, two stable states coexist, and a hysteresis loop becomes distinguishable at a constant frequency below (for softening type) or above (for hardening type) the critical frequency at which the bifurcations start to occur. Since two stable states are attainable due to hysteresis, changing the amplitude of the external force enables switching between these states for memory operations^3,34^. In addition, the oscillations in these two states after switching in between can be sustained with the same excitation force amplitude. The insets of Fig. 2a and Fig. S4a display the hysteresis responses of the microcantilevers under sweep-up (solid line) or sweep-down (dashed line) settings of the piezo actuator voltage at constant frequencies of 15.688 and 42.379 kHz, respectively.

To investigate the piezo actuator-based switching behavior of the cantilever with a softening nonlinearity, a constant V_Piezo_ = 28 mV (rms) signal with a frequency of 15.688 kHz was applied to the piezo actuator. Then, the steady-state V_Piezo_ excitation signal was increased or decreased by 8 mV for a duration of 100 ms to switch between the stable states of the microcantilever. Figure 2b demonstrates the piezo actuator-based memory operation performances of the GaN microcantilevers, where the top panels show the variation in V_DS_ (proportional to oscillation amplitude), and the bottom panels show the ±8 mV V_Piezo_ triggers for 100 ms around the mean value of 28 mV. Figure S4b shows similar switching behavior between stable states triggered by V_Piezo_ = ±6 mV, around the mean value of 157 mV for a duration of 500 ms. We note that higher piezo actuator biases were required for the cantilever with dimensions 150 × 50 μm to operate in the nonlinear regime since the critical amplitude to drive the microcantilever in the hysteresis region is higher.

### Memory operations utilizing photoacoustic excitations

**Fig. 3:**
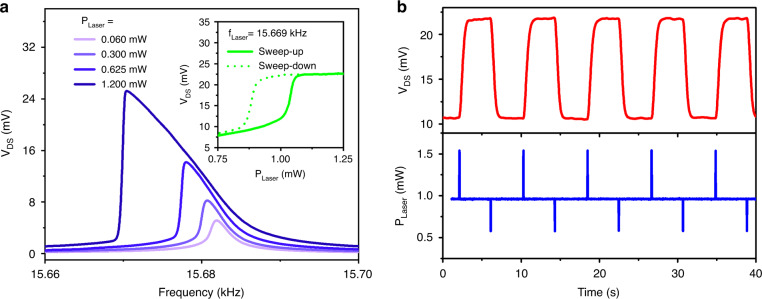
Memory operations using photoacoustic excitation by a pulsed laser at 520 nm. **a** Softening-type nonlinearities exhibited by the GaN microcantilever for different laser powers. The inset shows the hysteresis behavior at a frequency of 15.669 kHz. **b** Photoacoustic switching operations of the GaN microcantilever. A laser power of 0.9 mW was used to keep the cantilever in the bistable region, while differential power of 0.6 mW (up) and 0.4 mW (down) was used to make the transitions. Because of the softening-type intrinsic properties, all resonance curves in (**a**) were swept from high to low frequencies (sweep-down)

In addition to piezo actuator-based excitation, GaN microcantilevers can be excited through photoacoustic waves generated near the cantilever base using a pulsed laser^36,37^. Pulsed laser irradiation-induced periodic heating and cooling of the localized region results in expansions and contractions, which create acoustic waves in the structure. Hence, these pulsed laser-based excitations are called photoacoustic excitations. Details of the photoacoustic generation in the microcantilever are reported elsewhere^29,31,36^. A pulsed 520 nm laser was focused near the cantilever HFET using an X-Y-Z micropositioner, as shown in Fig. S5, and the resonance characteristics of the microcantilevers were recorded at different power settings. The results are presented in Fig. 3a and Fig. S4c, where the softening and hardening characteristics of microcantilevers, similar to the piezo actuator-based excitations, can be clearly seen beyond a certain laser power (0.3 and 0.7 mW, respectively). Keeping the laser pulsing frequency constant and sweeping the laser power higher and lower resulted in a similar hysteresis response as those obtained with the piezo ac bias sweep; the results are shown in the insets of Fig. 3a and S3c. Taking advantage of these hysteresis characteristics, multiple memory switching operations were demonstrated at a fixed frequency of 15.669 kHz by changing the laser power for 30 ms to switch on/off the microcantilever, as shown in Fig. 3b. The laser with a 520 nm wavelength was kept at a base power of 960 μW to operate the microcantilever in its hysteresis regime. The laser power was increased by 580 μW for 30 ms to reach the high state from the low state, while a 380 μW reduction in laser power resulted in switching from the high to the low state. Figure S4d shows laser-based memory operations using the microcantilever with hardening characteristics, where the operations were performed with a base laser power of 550 μW and switching was achieved by changing the power up and down by 500 and 450 μW, respectively, for ~100 ms.

**Fig. 4:**
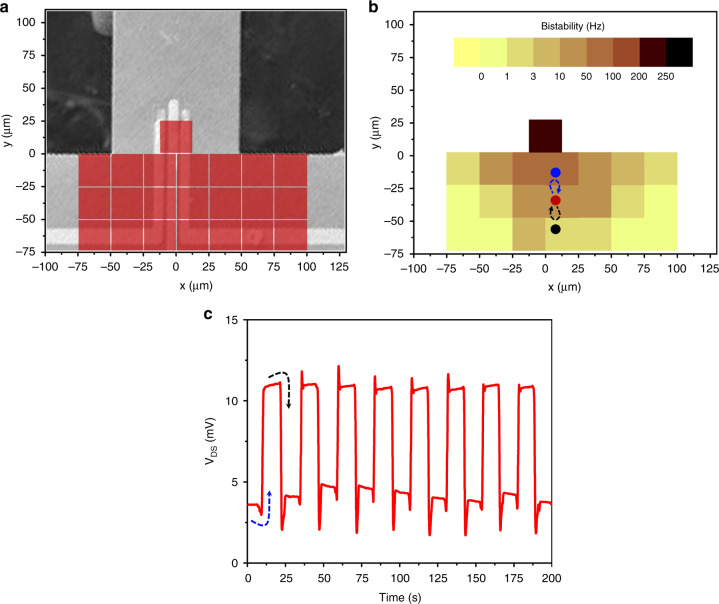
Switching the microcantilever oscillation state in the bistable region by changing the laser excitation location. **a** Red squares indicate the grids for the laser beam locations used for bistability mapping, superimposed on the SEM image of the cantilever under study. **b** Bistability map of the frequency gap (between up transition and down transition) of the photoacoustically excited microcantilever. As the laser location approaches the cantilever HFET (higher excitation), the bistability frequency gap increases. **c** Switching the cantilever repeatedly by manually adjusting the laser locations away from the cantilever base. To go from the low to high state, the laser beam focused on the cantilever surface, indicated by the red dot in (**b**), was moved to the higher bistable region (blue dot) for a second and brought back to its original position, as shown by the blue arrow in (**b**). The same moving procedure to the lower bistable region (marked by the black dot) was performed to switch the cantilever off, as shown by the black arrow in (**b**)

For photoacoustic-based excitations, the location of the focused beam is crucial to reveal microcantilevers’ intrinsic nonlinearities. The bistable regime, defined as the frequency difference between drop and jump frequencies (due to the hysteresis between forward and backward resonance curves), was mapped by measuring the resonance curves at various locations on and near the base of the cantilever, as illustrated in Fig. 4a. An SEM image of the cantilever with dimensions 250 × 100 µm is used as background, and the center points of highlighted squares are ~25 µm apart from each other. Figure 4b demonstrates the bistability measurements of the microcantilever. The highest bistability (~230 Hz) was observed when the laser beam was focused on the microcantilever HFET. Moving the laser beam away from the cantilever base reduces the bistable region as the effective amplitude of the acoustic waves reaching the cantilever decreases with increasing distance from the cantilever base. This dependence of the bistability on the laser beam location can also be used to demonstrate memory operations. Figure 4c displays switching between the cantilever states in the bistable regime utilizing different laser beam locations. The laser beam was first focused near the cantilever with some bistability for steady-state operations, as illustrated with the red dot in Fig. 4b. To switch the cantilever from the low to the high state, the laser beam was manually moved for 1 s toward the microcantilever HFET, where the bistability is higher, as shown by the blue arrow in Fig. 4b. On the other hand, when the laser beam was moved for 1 s to another position away from the microcantilever, as shown by the black arrow in Fig. 4b, the cantilever switched from the high to the low oscillatory state. Fig. S6 shows laser location-based memory operations utilizing hardening-type nonlinearities of the microcantilever with dimensions of 150 × 50 μm.

### Combining two piezo actuator excitations to switch microcantilever oscillation

**Fig. 5:**
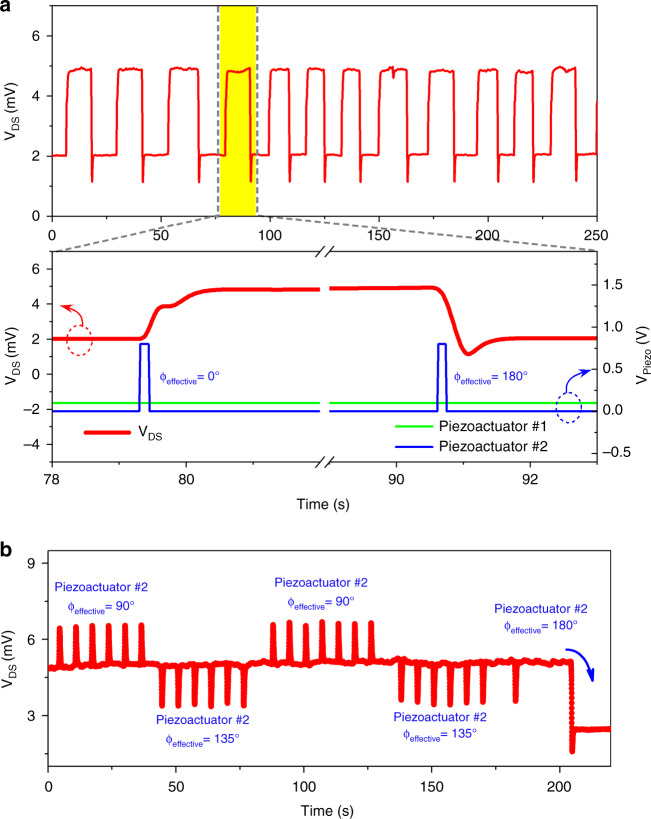
Switching the microcantilever oscillatory states using constructive and destructive interference from two excitations. **a** Memory operations utilizing two identical piezo actuators when piezo actuator #1 was attached directly under the microcantilever, while piezo actuator #2 was positioned ~2 cm away from the microcantilever. Piezo actuator #1 (15.974 kHz, 100 mV) was used to maintain microcantilever oscillations in the bistable region, while piezo actuator #2 (15.974 kHz, 800 mV) was employed to change the cantilever’s current state. To magnify the surface acoustic wave reaching the microcantilever to switch from the low state to the high state, the effective phase of the bias applied to the second piezo chip (φ_effective_) was set as 0° relative to the first piezo actuator signal. To reset the cantilever state, an effective phase of 180° relative to the first piezo actuator signal was employed. **b** Effects of the different effective phases of the piezo actuator #2 bias on the cantilever current state. The signals with 90° and 135° effective phase differences did not change the cantilever status from the high oscillatory state, as the differential excitation energy was not enough to enable phase transition

Following demonstration of memory operations separately through the piezo actuator and photoacoustic-based excitation of the GaN microcantilevers with softening and hardening nonlinearities, we examined bistable memory operations with two excitation sources—a primary and a secondary source. As mentioned earlier, a single excitation source (piezo actuator or laser) is not very efficient for developing a mechanical memory where multiple resonators need to be separately switched back and forth between the bistable states independently. To overcome this limitation, surface waves created by these two excitation sources (piezo actuator-based and photoacoustic-based) can be optimized to exploit constructive and destructive interference. To verify this concept, we first used a second piezo actuator attached to a printed circuit board (PCB) on which the first piezo actuator with the microcantilever (250 × 100 μm) was placed. The first piezo actuator was attached directly under the microcantilever, while the second piezo actuator was positioned ~2 cm away from the microcantilevers (see Fig. S7). The first (primary) piezo actuator was used to maintain the cantilever oscillation in the bistable regime, while the second piezo actuator was employed to switch between the microcantilever states by adding or removing acoustic power through constructive or destructive interference. Both provided excitation at 15.974 kHz. Figure 5a demonstrates the memory operations using two piezo actuators. The second piezo actuator was turned on for 100 ms (duration of gate pulse) to change the cantilever’s current status. To switch from the low state to the high state, the effective phase of the signal applied to the second piezo chip (ϕ_effective_) was kept at 0° relative to the first piezo actuator signal. Therefore, the magnitude of surface waves reaching the microcantilever was enhanced due to constructive interference. On the other hand, to change the cantilever state from high to low, an effective phase of 180° relative to the first piezo actuator was utilized, which resulted in destructive interference and a reduction in the overall excitation. We also used different effective phases to verify the concept of constructive and destructive interference of two excitations. As seen from Fig. 5b, the signals with 90° and 135° phase differences did not change the cantilever status from the high oscillatory state, as sufficient reduction in the overall excitation was not achieved for these phases.

### Switching enabled by a combined piezo actuator and photoacoustic excitation

**Fig. 6:**
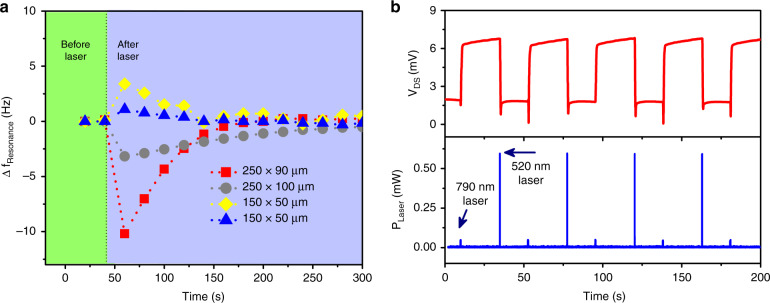
Utilizing the thermal effects for mechanical switching. **a** Shift in the resonance frequency as a function of time due to heating the microcantilever using a continuous laser. The microcantilevers were heated for 0.5 s. Due to heating, the resonance frequency temporarily switches to higher or lower frequencies depending on the cantilever dimension. **b** Switching the cantilever with heating effects (to switch on) and destructive interference (to switch off). The microcantilever with dimensions 250 × 100 μm was maintained in the bistable regime using the piezo actuator biased with a constant V_Piezo_ of 44 mV ac at 15.684 kHz. The 790 nm laser (~50 μW) was turned on for 300 ms (duration of gate pulse) to switch the cantilever ON, while the 520 nm laser was pulsed at a frequency of 15.684 kHz (with a phase of 180°) and a pulse width of 50 ms (duration of gate pulse) to turn the cantilever OFF. The switch-on operation is driven solely by heating effects, while destructive interference was utilized for the switch-off operation

The second piezo actuator that was used for switching can be replaced with photoacoustic excitation induced by a pulsed laser. However, exposing nearby locations of a microcantilever HFET to the laser can cause shifts in the fundamental frequency due to localized thermal effects. Such effects can be clearly seen in Figs. 2a and 3a of the low amplitude resonance curves. While the resonance frequency of the microcantilever recorded under excitation from the piezo actuator (biased at 10 mV rms) was ~15.690 kHz, the resonance frequency measured for the photoacoustically excited microcantilever (with 60 μW laser power) was certainly below 15.690 kHz, indicating a resonance shift. Alterations in the resonance frequencies of the GaN microcantilevers with dimensions of 250 × 90 μm, 250 × 100 μm, and two dimensions of 150 × 50 μm after 520 nm laser exposure for ~500 ms are shown in Fig. 6a. We find that microcantilevers demonstrating softening-type intrinsic nonlinearities (250 × 90 μm and 250 × 100 μm) exhibit a reduction in resonance frequency, while the resonance frequencies of the microcantilevers with hardening-type intrinsic nonlinearities (150 × 50 μm) shift to higher frequencies after exposure to the laser. To further verify these temperature-related resonance shifts, a microcantilever with dimensions 250 × 100 μm was attached on top of a ceramic heater. The ceramic heater was turned on for 3.5 and 5 seconds to provide pulsed heating, and the resonance shifts were subsequently measured. As seen in Fig. S8a, the resonance frequency initially decreased as the microcantilever temperature continued to rise slowly following the initial heating, reaching the lowest value of −2.2 Hz at ~110 s and then recovered slowly back to its initial value as the temperature rise in the cantilever subsided. Although at first glance, this temperature change might be perceived as an undesirable side effect of any potential laser-based excitation, in reality, this resonance shift due to heating can be utilized to perform memory operations for both hardening and softening types of microcantilevers under study. This is because as the resonance frequency decreases for microcantilevers with softening characteristics, the bistable regime also shifts equivalently. Thus, if the microcantilever oscillates at its lower branch of the bistable region, then the deflection amplitude moves to the oscillatory state due to heating, as the operating frequency is no longer in the bistable region of the cantilever. As the cantilever cools down, the state of the cantilever remains in the high state of the bistable regime since it was already in the oscillatory state due to the heating effect. The cantilever switching effect caused by a heat pulse is shown schematically in Fig. S8b. This was also verified experimentally using the setup illustrated in Fig. S9, and the results are shown in Fig. S8c, where following the initial 3.5 s heat pulse provided by the heater (between 8 and 12 s), the amplitude of the cantilever kept rising slowly until it suddenly jumped into the high oscillatory state at ~45 s and then remained there until it was brought down to the low oscillatory state by the second piezo actuator providing excitation at 180 phase difference with the first piezo actuator.

Utilizing the thermal effects and destructive interference, memory operations with a piezo actuator as the primary source of excitation and two lasers (520 nm and 790 nm) providing photoacoustic excitation, as well as the associated heating effect, are experimentally demonstrated in Fig. 6b. The experimental schematic is shown in Fig. S10. A low-power (~50 μW) laser with a 790 nm wavelength was focused on the microcantilever HFET, while the 520 nm laser (600 μW) was carefully focused ~100 μm away from the cantilever base to ensure negligible thermal effects. Even though the power of the 790 nm laser was low, it was able to shift the resonance frequency of the microcantilever (see Fig. S11). The microcantilever with dimensions 250 × 100 μm was maintained in the bistable regime using the piezo actuator biased with a constant V_Piezo_ of 44 mV ac at 15.684 kHz. To change the cantilever current state from low to high, the 790 nm laser (steady, no pulsing) was kept on for 300 ms (duration of gate pulse) to heat the cantilever. This caused a shift in the oscillatory status of the cantilever from low to high. On the other hand, the 520 nm laser with a pulsed frequency of 15.684 kHz and 180° phase shift relative to the piezo actuator signal was kept on for a 50 ms gate pulse duration to make the cantilever switch from the high state to the low state by destructive interference (as discussed above regarding the second piezo actuator-based excitation). The lasers were exchanged for the microcantilever with dimensions of 150 × 50 μm. A 520 nm laser (focused on the cantilever HFET) was employed to change the cantilever oscillatory status from low to high utilizing the thermal effects, while destructive interference-based switching from the high to low state was achieved using a 790 nm laser focused ~100 μm away from the cantilever. The results are shown in Fig. S12.

### Enhancing photoacoustic excitation with plasmonics to reduce switching power

**Fig. 7:**
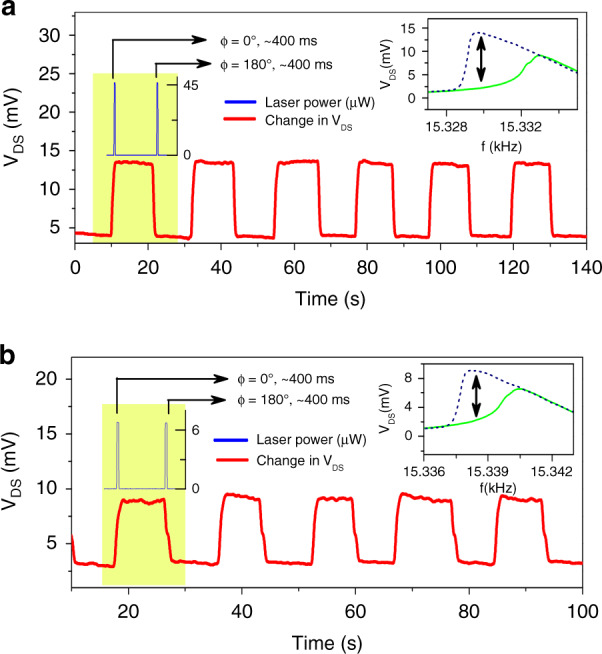
Effect of 1.5 nm gold plasmonic layer deposition on the photoacoustic state transition in the microcantilever. **a** Before 1.5 nm gold deposition, a 790 nm laser with a power of 45 µW (for 400 ms gate pulse duration) at phases 0 and 180° was employed to switch the cantilever state from OFF to ON, and vice versa, while the microcantilever was excited using the piezo actuator at 34 mV bias. **b** After 1.5 nm of gold deposition on the cantilever surface, only 6 µW laser power was required to switch between the cantilever bistable states. Insets show the forward (green) and backward (blue) resonance curves of the cantilever at the piezo bias used in the switching operations. High and low states are shown with black double-sided arrows, corresponding to operational frequencies of 15.330 and 15.338 kHz for (**a**) and (**b**), respectively

Optimizing the power and the beam location of the lasers is necessary not only to dilute the thermal effects but also to switch efficiently between the bistable states, expending the lowest possible energy. Figure 7a shows the switching characteristics of the microcantilever with a width of 100 μm. Shifts in the resonance frequency due to thermal effects were avoided by using the low-power 790 nm laser focused slightly away from the cantilever HFET. The 790 nm laser with a pulse width of 400 ms, frequency of 15.330 kHz, and power of 45 μW was operated under constructive and destructive interference settings to change the cantilever current state, while the piezo actuator was biased at a frequency of 15.330 kHz and V_Piezo_ = 34 mV was utilized as the primary excitation source to sustain the bistable oscillations (see Fig. 1b for experimental diagram). The inset of Fig. 7a displays forward and backward sweeps. To reduce the required switching laser power of 45 μW, the absorption properties of the surface can be improved, which would then enhance the amplitude of the photoacoustic waves. Very significant enhancement in photon absorption and enhancement in the photoacoustic signal is possible by a thin Au nanoparticle coating on the surface, which has been observed to amplify the photoacoustically generated signal by up to two orders of magnitude^37,40^. Accordingly, we deposited 1.5 nm Au on the top surface of the entire chip containing the GaN microcantilevers to reduce the laser power required for switching. The resonance frequency of the microcantilever was increased by ~6 Hz following Au nanoparticle deposition, as the surface stress changed significantly, overcoming any mass loading effect, in agreement with our earlier observations^31^. Following Au nanoparticle (NP) deposition, switching between states utilizing the 790 nm laser could be realized with a significantly reduced power of 6 μW (compared to 45 μW prior to Au deposition), as observed from the inset (left) pulse magnitudes of Figs. 7a, b. The right inset of the figures shows the hysteresis region between the forward and backward curves, which can be seen to be approximately the same before and after gold deposition. However, the amplitude difference between the low and high states is lower than the difference before Au coating since a lower V_DS_ (0.22 V compared to 0.28 V before, with V_GS_ = −2.6 V and a constant I_DS_ = 100 µA) was measured after Au deposition, possibly due to minor alterations in the conductivity of the HFET due to Au NP deposition (the HFET and resonance characteristics before and after Au NP deposition are shown in Fig. S13). Therefore, the sinusoidal changes in V_DS_ are also lower following Au deposition.

**Fig. 8:**
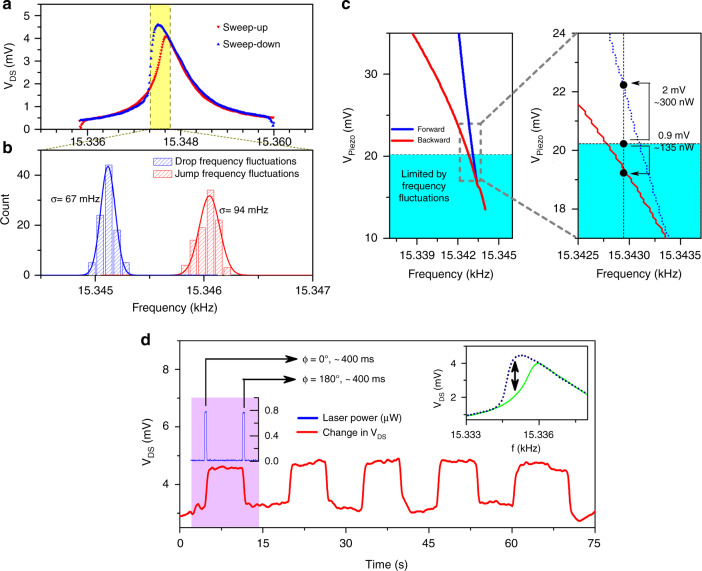
Theoretical and experimental demonstration of the minimum laser power required to switch between the high (ON) and low (OFF) states of microcantilever oscillation. **a** Typical sweep-up and sweep-down resonance characteristics of the microcantilever with excitation from a piezo actuator at a bias of 24 mV. **b** Transition frequency distribution around the “OFF to ON” (shown by blue bars) and “ON to OFF” (shown by red bars) determined for 100 transitions each. Each distribution was fitted with Gaussian curves to yield standard deviations σ of 67 and 94 mHz. **c** Left panel: A bistable frequency plot was generated from Equations S6 and S7, as discussed in the supplemental section, with the upper white region >95% confidence interval (frequency difference of 4 times averaged σ) in switching transitions. Right panel: Magnified section of the bistable frequency plot (dotted rectangle) shows the differential V_Piezo_ values needed to make the ON and OFF transitions, which are 2 and 0.9 mV, respectively. The corresponding laser estimation is also shown as 300 and 135 nW. **d** Switching the microcantilever using the 790 nm laser set at 800 nW power. The inset shows the forward (green) and reverse (blue) resonance curves of the cantilever at the piezo bias (20 mV ac rms) used to perform the switching experiments. The high and low states of 2.1 and 4.3 mV are shown with the double-headed arrow at a resonance frequency of 15.335 Hz

To estimate the lowest energy needed to make the transition from a nonoscillatory to an oscillatory state and vice versa, the minimum piezo actuator amplitude to drive the microcantilevers in the bistable regime must be calculated. Therefore, we first estimated the nonlinear cubic constant (α) from experimental piezo actuator-based forward and backward curves using the modified Duffing equation discussed in detail in Supplementary Discussion I. The minimum piezo actuator bias to excite the cantilever (dimensions of 250 × 100 μm) with bifurcations was calculated to be ~13.5 mV, as discussed in Supplementary Discussion I, using the estimated cubic constant. The critical bifurcation frequency where drop and jump frequencies compose an inflection point is predicted to be 15.344 kHz at this excitation voltage using the Duffing equation^33,34^. However, exciting the microcantilever at the critical frequency will very likely result in noise-assisted stochastic switching between the states^25^. Frequency fluctuations due to external and internal sources, including thermomechanical noise^41^ and damping noise^42^, considerably affect the stability of the cantilever at its current state^43^. Thus, consistent memory operations can only be performed while exciting the microcantilever at marginally lower Δ*f* (depending on the instrument and noise limitations) below the critical frequency, since softening nonlinearities are dominant for this cantilever. Hence, frequency fluctuations at drop and jump frequencies were measured at a piezo actuator bias of 24 mV, which exceeds the critical amplitude of 13.5 mV to obtain a clear difference between bifurcation frequencies, with reference to the hysteresis curve shown in Fig. 8a. We considered 100 cases of jump-up and jump-down transitions, and the frequency distributions are plotted in Fig. 8b. There are two histograms that were separately fit with Gaussian curves, which exhibited standard deviations (σ) of 67 mHz and 94 mHz for drop and jump frequency fluctuations, respectively. The bistable regime of the microcantilever as a function of the piezo actuator bias V_Piezo_, defined as the region between bifurcation points (drop and jump frequencies), was numerically calculated using the estimated value of α (nonlinearity parameter) = −9.20 × 10^6^, using Equations S6 and S7 in the supplementary discussion, and the result is shown in Fig. 8c. To avoid frequency fluctuations for the sake of stability, the frequency difference between drop and jump frequencies needs to be ~4σ to assure a 95% confidence interval. The 4σ frequency interval was calculated by averaging the standard deviations for jump and drop frequency fluctuations, which yielded 4((67 + 94)/2) mHz = 0.322 Hz. The minimum operation V_Piezo_ bias satisfied the 95% confidence interval (0.322 Hz frequency difference between the blue (jump frequency) and red (drop frequencies) lines in Fig. 8c) for memory operations is 20.2 mV, which is shown by the dotted line in Fig. 8c. The operation frequency was computed as 15.3429 kHz, which is the mean of the drop and the jump frequencies at V_Piezo_ = 20.2 mV determined from the theoretically calculated bistability diagram. From Fig. 8c, we find that to perform switching operations at the center frequency of 15.3429 Hz, a 2 mV increase in V_Piezo_ is required to switch from the low to high state, while a 0.9 mV reduction in V_Piezo_ is necessary to make the transition from the high state to the low state, as shown in Fig. 8c. The equivalent laser powers corresponding to these calculated V_Piezo_ biases (i.e., 2 mV to set to high and 0.9 mV to reset to low) can be estimated by comparing the resonance amplitudes of the photoacoustic and piezo actuator-based excitations, as discussed below.

Direct comparison of the microcantilever resonance amplitude caused by the piezo actuator and photoacoustic excitation can offer a reliable estimate of the conversion factor since the deflection sensitivity of the microcantilever HFET does not change with the excitation source, even though laser-based photoacoustic excitation may shift the resonance frequency slightly^36^. Comparing the experimental resonance characteristics caused by the two excitation techniques (plotted in Fig. S14), we find that the resonance amplitude resulting from photoacoustic excitation with an 800 nW laser (790 nm) will be equivalent to the resonance behavior obtained from a piezo actuator bias V_Piezo_ = 5.0 mV. Then, in Fig. 8c, the V_Piezo_ bias increase of 2 mV as needed to reach the “high” oscillatory state would be equivalent to operating the laser with ~300 nW power in the constructive interference setting (primary excitation still being the piezo actuator). On the other hand, V_Piezo_ can be decreased by 0.9 mV, or equivalently by a laser power of ~135 nW under destructive interference conditions, to reach the “low” oscillatory state. Taking the higher of these two as the power to make a reliable transition, we determine 300 nW as the minimum power of the 790 nm laser at this specific laser beam location for memory operations.

For experimental verification of the lower power limit determined above for memory operations, we reduced the laser power to 800 nW to the minimum value possible, limited by the instrument and the measurement setup. The 790 nm laser was carefully focused near the microcantilever HFET to obtain the highest resonance amplitude. Switching operations were performed with an 800 nW laser (with primary excitation from the piezo actuator biased at 20 mV ac, rms), which was turned on and off for 400 ms, with phases of 0 and 180° to make transitions from low to high and vice versa. The results are shown in Fig. 8d. The inset shows the forward (green) and backward (blue) resonance curves of the cantilever at a piezo bias of 20 mV. From Fig. 8d, reliable bistable switching operations can be observed using 800 nW laser power, much above the noise limit, supporting the validity of our theoretical estimate. Further reduction in the minimum laser power required for switching operations can be achieved by selecting a more appropriate laser wavelength that is better absorbed in the substrate or through higher plasmonic amplification, which can lead to higher photoacoustic excitation.

The minimum laser power determined via theoretical and experimental means, as discussed above, is only an external estimate limited by the experimental and instrument setup. It does not provide a reliable estimate of the actual energy required by the microcantilever to switch from one state to another. Indeed, only a very small fraction of the energy provided by the 790 nm laser is absorbed by the GaN microcantilever since a large fraction is reflected back (especially when the laser is focused on the HFET with metal contacts), and from the remainder, only very little is absorbed by the material, as the bandgap of GaN (~3.42 eV) is much larger than the photon energy at 790 nm. The absorption of optical energy is mostly facilitated by plasmonic absorption^37^, which is difficult to estimate accurately. Therefore, we calculated the switching power/energy utilizing acoustic wave equations starting from an estimation of the cantilever oscillation amplitude. The amplitude of the photoacoustic wave generated with an 800 nW laser at the microcantilever base is first estimated using the conversion factor. We note that the photoacoustic waves generated with the 790 nm laser, operating at a power of 800 nW, caused a similar response generated by the piezo actuator excitation at V_Piezo_ = 5 mV, and since the laser beam was focused on the cantilever fixed end, the amplitude of the base displacement can be assumed to be equal to the piezo actuator displacement at a 5 mV bias. The displacement at a bias of 5 mV ac rms is 110 pm using the following relationship, i.e., piezo actuator displacement (δ_Piezo_) = G_Piezo_ V_Piezo_, where G_Piezo_ and V_Piezo_ are the displacement coefficient of the piezo actuator (2.2 × 10^−8 ^m/V provided by the manufacturer) and the amplitude of the ac bias (5 mV), respectively. Utilizing a plane progressive acoustic soundwave assumption, the acoustic power (P_ac_) can be calculated using the equation^39,44^, *P*_*ac*_ *=* *δ*^*2*^ *ω*^*2*^ *Z A*, where δ is the particle displacement and ω and A are the excitation frequency and the area of the laser exposure (~20 µm diameter circle at the focal point, provided by laser manufacturer WorldStar Tech), respectively. Z is the acoustic impedance of GaN, calculated using *Z* *=* *p c*, where ρ is the density of GaN (6.15 × 10^3^ kg/m^3^)^45^ and c is the sound velocity in GaN (8000 m/s)^45,46^, which yields Z = 49.2 × 10^6 ^kg m^−2^s^−1^. Using the values of Z, ω = 96.36 kHz, and the area of the laser exposure, in the equation for acoustic power P_ac_, we determine that the switching power needed to be 1.74 × 10^−12 ^W, which corresponds to an acoustic energy of 7 × 10^−13 ^J considering a 400 ms exposure time.

## Discussion

In general, memory and logic operations based on resonators are operated in dynamic mode taking advantage of resonance and nonresonance modes^3^. Among different techniques switching from on-resonance to off-resonance behaviors, a significant amount of work has been reported on mechanical memory and logic operations in the nonlinear regime, taking advantage of the existence of two different stable oscillatory states at the same frequency (bistability)^3^. A comprehensive list of memory and logic operations reported in the literature utilizing MEMS and NEMS, including double clamped beams, nano and microcantilevers, and comb drives, is presented in Supplementary Table II to put the current work in perspective with the best results in this area. With regard to the performance analysis of memory devices, the power/energy requirement per switching cycle is an important parameter to consider, in addition to size and operational complexities. From Supplementary Table II, we find that the lowest reported energy consumption for electrostatically actuated doubly clamped nanobeams and nanocantilevers scaled down to the range of 10^−11^ – 10^−17 ^J^7,14,18,19,47^, which is very comparable to memory devices based on CMOS transistor technology, which is typically in the range of 10^−16^ – 10^−18 ^J^3^. We note that the minimum switching energy for our GaN microcantilevers through photoacoustic excitation is ~10^−13 ^J, which, although low, is still several orders of magnitude higher than the lowest switching energy reported for NEMS devices. This is expected, however, since the size and mass of our GaN microcantilevers are much larger than those of nanoresonators with reported ultralow power performances^3,19,47^. To account for this variation, we have included a column in Supplementary Table II showing calculations of switching energy per unit mass per cycle for the selected resonators. Normalization was performed by simply dividing the required switching energy by the resonator mass. The normalized switching energy per mass of the GaN microcantilevers is on the order of 10^−3 ^J/kg, which is very comparable, if not better than the best reported experimental results in the literature, spanning various length scales, excitation techniques, and readout schemes.

Another notable feature presented in the table is the readout methodology used in mechanical memory and logic operations. Optical transduction methods were generally employed to readout the position of the resonators. Transducing tip oscillations directly into electrical signals using an embedded HFET offers a significant advantage for our system since the usage of optical deflection transduction schemes makes the system complex and cumbersome, especially when applied to an array of resonant devices. In addition to the readout methodology, the excitation source of the resonator is a key factor in memory/logic operations performed in a nonlinear regime. Electrostatic actuation is commonly used since an electrostatic force can be easily applied to the system, and it can be used as a sensing mechanism. The required switching energy with the electrostatic actuation method is quite small for memory and logic operations because of the extremely small capacitive term in the formulation^14,19^. However, the main drawback of electrostatic actuation is the requirements of large ac and dc voltages (tens of V) to reach the nonlinear regime. The required signals to drive GaN microcantilevers in their nonlinear regime are considerably lower with the piezo actuator (mV regime) and photoacoustic-based excitation methods. In addition, photoacoustic-based switching offers many advantages in mechanical memory operations, including position-controlled switching, which is truly unique among all competing excitation techniques and is readily applicable to array-based operation. In addition, electrostatic excitation introduces additional spring softening effects into the resonance characteristics, which are typically used to manipulate intrinsic nonlinearities^43,48–50^. As an alternative to regulating the nonlinear characteristics with electrostatic actuation, both softening and hardening nonlinearities are readily accessible with GaN microcantilevers with different dimensions^34^.

In addition, utilizing two different phase-controlled actuation methods in dynamic memory operations, i.e., the piezo actuator-based primary excitation to oscillate the microcantilever in the bistable regime and the photoacoustic secondary excitation to switch between the stable states, underlines the versatility of our system and its applicability for resonator arrays. This phase-controlled dual-mode excitation can unlock the true potential of these resonators for practical applications through the realization of complex mechanical memory operations and computation. This is because multiple resonators with similar resonance and nonlinearity properties can be assembled on the same piezo actuator, and a tracking system can be used to move the laser beam with a microscale resolution to set or reset the microcantilever oscillatory states as desired. In the case of a single external actuation force-based memory and logic operations, controlling each resonator requires separate excitation and detection mechanisms, which can make the process quite cumbersome.

Another important factor to be considered in MEMS/NEMS-based dynamic memory and logic operations is the switching rate, which is limited by the rise and fall time^14,51^. The theoretical switching rate is given by Γ *=* *f*_*o*_*/Q*^3,8^, where f_0_ and Q are the resonance frequency and quality factor of the resonator, respectively. The estimated operation rates are 1.625 s and 0.11 s for the GaN microcantilevers with dimensions of 250 × 100 μm and 150 × 50 μm, respectively. The ambient pressure around the microcantilever can be changed to control the quality factors of the resonators and optimize the switching rate. Raising the pressure, which reduces the quality factor, not only improves the switching rate but also decreases the frequency fluctuations^41^, which will reduce the minimum laser power to switch the cantilever status. However, a higher microcantilever excitation will be needed to operate them in the nonlinear regime at higher pressure.

In summary, we have demonstrated for the first time dynamic memory operations involving bistable states in GaN piezotransistive microcantilevers operated in their intrinsic nonlinear regime. A novel multimodal excitation scheme involving a piezo actuator and a laser, acting as primary and secondary excitation sources to perform constructive and destructive interference based on their phase relationships, was used to demonstrate hardening and softening nonlinearities in these cantilevers and switch between the high and low oscillatory states in the bistable regime, respectively. In addition to phase, variation was also demonstrated to be an alternative method to perform repeatable switching, which is unique to the photoacoustic method among all other methods reported in the literature. The minimum energy required for reliable memory operations utilizing secondary photoacoustic excitation was determined to be less than a picojoule, utilizing plasmonic amplification of the signal by Au nanoparticles deposited near the cantilever, which resulted in a several-fold increase in the photoacoustic signal magnitude. When normalized for cantilever mass and resonance frequency, the switching energy was estimated to be one of the lowest reported thus far for oscillators with various dimensions, excitation sources, and readout schemes.

## Materials and methods

### Device structure and fabrication

A high-quality epitaxial wafer, which consists of 20 nm Al_x_Ga1-xN (x = 0.25), 1 μm GaN, 0.3 μm buffer layer, and 675 μm Si layers, was purchased from NTT Advanced Technology Corporation. AlGaN/GaN HFET-embedded GaN microcantilevers were fabricated at the Institute for Electronics and Nanotechnology at the Georgia Institute of Technology. A brief fabrication procedure is as follows: first, AlGaN and GaN layers are etched using the Plasma-Therm inductively coupled plasma (ICP) tool to define the HFET and microcantilever areas, respectively. After deposition of the metal stack of Ti (20 nm)/Al (100 nm)/Ti (45 nm)/Au (55 nm) using a CHA e-beam evaporator, HFET ohmic contacts were formed with a rapid thermal annealing process (SSI RTP). Schottky-metal contacts of Ni (25 nm)/Au (375 nm) and the probe metal stack of Ti (20 nm)/Au (150 nm) were deposited using the e-beam consecutively. Finally, deep Si etching with the Bosch process (STS ICP) was used to release the microcantilevers. Au layers for plasmonic amplification were deposited using an electron beam deposition system (CCS CA-40 e-beam evaporator).

### Characterization

The fabricated microcantilevers were placed on a linear piezo actuator (PL055 from Physik Instrumente) with dimensions of 5 × 5 × 2 mm. For electrical connection, the piezo actuator and the cantilevers were attached to a custom-designed printed circuit board (PCB), and the HFET probe contacts were wire-bonded to the PCB. A constant drain current (I_DS_) of 100 μA was supplied to the cantilever HFET under study using a source measurement unit (Keysight 2902 A), while the gate was biased at an appropriate gate voltage. Two temperature-compensated pulsed laser systems with wavelengths of 520 and 790 nm, purchased from WorldStar Tech, were employed for photoacoustic excitation. Sinusoidal changes in the drain-source voltage of the HFET were measured using a lock-in amplifier (Stanford Research Systems, SR850). For switching experiments with multiple excitation sources, two signal generators (Keysight 33210 A) were used to pulse the laser(s) and the piezo chip. The optical power of the laser used in our experiments was measured at the same pulsing settings and conditions using a photodiode (Newport 880-SL) and an optical power meter (Newport 1918-R) immediately after the switching experiment. All the characterization and switching experiments were carried out at room temperature and at ~1 mTorr pressure in a homemade vacuum chamber (fitted with a two-stage mechanical/turbomolecular pump) with optical viewports and electrical feedthrough.

## Supplementary information

Supplementary Information

## References

[1] Swade, D. & Babbage, C. In *Difference engine: Charles Babbage and the quest to build the First Computer* (Viking Penguin, 2001).

[2] Rueckes T (2000). Carbon nanotube-based nonvolatile random access memory for molecular computing. Science.

[3] Ilyas S, Younis MI (2020). Resonator-based M/NEMS logic devices: Review of recent advances. Sens. Actuators A: Phys..

[4] Lee TH, Bhunia S, Mehregany M (2010). Electromechanical computing at 500 degrees C with silicon carbide. Science.

[5] Kazmi SN (2017). Tunable nanoelectromechanical resonator for logic computations. Nanoscale.

[6] Badzey RL, Zolfagharkhani G, Gaidarzhy A, Mohanty P (2004). A controllable nanomechanical memory element. Appl. Phys. Lett..

[7] Mahboob I, Yamaguchi H (2008). Bit storage and bit flip operations in an electromechanical oscillator. Nat. Nanotechnol..

[8] Hafiz MAA, Kosuru L, Younis MI (2016). Microelectromechanical reprogrammable logic device. Nat. Commun..

[9] Villanueva L (2013). Nonlinearity in nanomechanical cantilevers. Phys. Rev. B.

[10] Moon FC, Shaw SW (1983). Chaotic vibrations of a beam with non-linear boundary conditions. Int. J. Non-Linear Mech..

[11] Eichler A (2011). Nonlinear damping in mechanical resonators made from carbon nanotubes and graphene. Nat. Nanotechnol..

[12] Turner KL (1998). Five parametric resonances in a microelectromechanical system. Nature.

[13] Guerra DN, Imboden M, Mohanty P (2008). Electrostatically actuated silicon-based nanomechanical switch at room temperature. Appl. Phys. Lett..

[14] Guerra DN (2010). A noise-assisted reprogrammable nanomechanical logic gate. Nano Lett..

[15] Noh H, Shim S, Jung M, Khim ZG, Kim J (2010). A mechanical memory with a dc modulation of nonlinear resonance. Appl. Phys. Lett..

[16] Uranga A (2013). Exploitation of non-linearities in CMOS-NEMS electrostatic resonators for mechanical memories. Sens. Actuators A: Phys..

[17] Yao A, Hikihara T (2014). Logic-memory device of a mechanical resonator. Appl. Phys. Lett..

[18] Hafiz MAA, Kosuru L, Ramini A, Chappanda KN, Younis MI (2016). In-plane MEMS shallow arch beam for mechanical memory. Micromachines.

[19] Chappanda K (2017). A single nano cantilever as a reprogrammable universal logic gate. J. Micromech. Microeng..

[20] Al Hafiz MA, Kosuru L, Younis MI (2016). Electrothermal frequency modulated resonator for mechanical memory. J. Microelectromech Syst..

[21] Mahboob I, Mounaix M, Nishiguchi K, Fujiwara A, Yamaguchi H (2014). A multimode electromechanical parametric resonator array. Sci. Rep..

[22] Onuta T, Wang Y, Long CJ, Lofland SE, Takeuchi I (2012). Dynamic state switching in nonlinear multiferroic cantilevers. Appl. Phys. Lett..

[23] Onuta T, Wang Y, Lofland SE, Takeuchi I (2015). Multiferroic operation of dynamic memory based on heterostructured cantilevers. Adv. Mater..

[24] Venstra WJ, Westra HJ, van der Zant, Herre SJ (2010). Mechanical stiffening, bistability, and bit operations in a microcantilever. Appl. Phys. Lett..

[25] Venstra WJ, Westra HJ, Van Der Zant, Herre SJ (2013). Stochastic switching of cantilever motion. Nat. Commun..

[26] Gao N, Zhao D, Jia R, Liu D (2016). Microcantilever actuation by laser induced photoacoustic waves. Sci. Rep..

[27] Koskinen V, Fonsen J, Roth K, Kauppinen J (2008). Progress in cantilever enhanced photoacoustic spectroscopy. Vibrational Spectrosc..

[28] Chamassi K (2019). Capacitive silicon micro-electromechanical resonator for enhanced photoacoustic spectroscopy. Appl. Phys. Lett..

[29] Khan, D., Li, H., Gajula, D., Bayram, F. & Koley, G. H2 detection using plasmonically generated surface photoacoustic wave in Pd nanoparticle deposited GaN Microcantilevers. *ACS sensors* (2020). 10.1021/acssensors.0c01181 32964707

[30] Wu Q (2018). Photoacoustic microbeam-oscillator with tunable resonance direction and amplitude. Opt. Commun..

[31] Khan D, Li H, Bayram F, Gajula D, Koley G (2020). Photoacoustic detection of H2 and NH3 using plasmonic signal enhancement in GaN microcantilevers. Micromachines.

[32] Tomberg T, Vainio M, Hieta T, Halonen L (2018). Sub-parts-per-trillion level sensitivity in trace gas detection by cantilever-enhanced photo-acoustic spectroscopy. Sci. Rep..

[33] Lifshitz R, Cross M (2008). Nonlinear dynamics of nanomechanical and micromechanical resonators. Rev. nonlinear Dyn. Complex..

[34] Bayram F, Gajula D, Khan D, Gorman S, Koley G (2019). Nonlinearity in piezotransistive GaN microcantilevers. J. Micromech. Microeng..

[35] Qazi M, DeRoller N, Talukdar A, Koley G (2011). III-V Nitride based piezoresistive microcantilever for sensing applications. Appl. Phys. Lett..

[36] Talukdar A (2015). Piezotransistive transduction of femtoscale displacement for photoacoustic spectroscopy. Nat. Commun..

[37] Khan D (2017). Plasmonic amplification of photoacoustic waves detected using piezotransistive GaN microcantilevers. Appl. Phys. Lett..

[38] Bayram F, Khan D, Li H, Hossain MM, Koley G (2018). Piezotransistive GaN microcantilevers based surface work function measurements. Jpn. J. Appl. Phys..

[39] Talukdar A, Qazi M, Koley G (2012). High frequency dynamic bending response of piezoresistive GaN microcantilevers. Appl. Phys. Lett..

[40] Gao N, Zhao D, Jia R, Zhang D, Liu D (2017). Plasmonic Microcantilever with Remarkably Enhanced Photothermal Responses. Sci. Rep..

[41] Roy, S. K., Sauer, V. T. K., Westwood-Bachman, J. N., Venkatasubramanian, A. & Hiebert, W. K. Improving mechanical sensor performance through larger damping. *Science* **360**, 10.1126/science.aar5220. Epub 2018 Jun 14 (2018). 10.1126/science.aar5220 29903939

[42] Maillet O (2018). Measuring frequency fluctuations in nonlinear nanomechanical resonators. ACS nano.

[43] Sansa M (2016). Frequency fluctuations in silicon nanoresonators. Nat. Nanotechnol..

[44] Rienstra SW, Hirschberg A (2004). An introduction to acoustics. Eindh. Univ. Technol..

[45] Levinshtein, M. E., Rumyantsev, S. L. & Shur, M. S. in *Properties of Advanced Semiconductor Materials: GaN, AIN, InN, BN, SiC, SiGe* (John Wiley & Sons, 2001).

[46] Rais-Zadeh M (2014). Gallium nitride as an electromechanical material. J. Microelectromech Syst..

[47] Wenzler J, Dunn T, Toffoli T, Mohanty P (2014). A nanomechanical Fredkin gate. Nano Lett..

[48] Agarwal M (2006). Optimal drive condition for nonlinearity reduction in electrostatic microresonators. Appl. Phys. Lett..

[49] Kacem N, Arcamone J, Perez-Murano F, Hentz S (2010). Dynamic range enhancement of nonlinear nanomechanical resonant cantilevers for highly sensitive NEMS gas/mass sensor applications. J. Micromech. Microeng..

[50] Tiwari S, Candler RN (2019). Using flexural MEMS to study and exploit nonlinearities: a review. J. Micromech. Microeng..

[51] Aldridge J, Cleland A (2005). Noise-enabled precision measurements of a duffing nanomechanical resonator. Phys. Rev. Lett..

