# Supplementary material for: Mechanical memory operations in piezotransistive GaN microcantilevers using Au nanoparticle-enhanced photoacoustic excitation
Source: Microsyst Nanoeng. 2022 Jan 24;8:8. doi: 10.1038/s41378-021-00330-6 (PMC8784537; doi:10.1038/s41378-021-00330-6)
Supplement: Supplementary file 1 — Supplementary Information [file 41378_2021_330_MOESM1_ESM.docx]

**Supplementary Information**

**Mechanical Memory Operations in Piezotransistive GaN Microcantilevers using Au Nanoparticle Enhanced Photoacoustic Excitation**

Ferhat Bayram^1^, Durga Gajula^2^, Digangana Khan^1^, and Goutam Koley^1^

*^1^Holcombe Department of Electrical and Computer Engineering*

*Clemson University, Clemson SC 29634*

*^2^School of Electrical and Computer Engineering*

*Georgia Institute of Technology, Atlanta, GA 30332*

**Supplementary Discussion I. Estimating cubic constant using Duffing equation**

Effective mass of the GaN microcantilever needs to be estimated before performing nonlinear modeling of the cantilevers. Because of the overhang of the microcantilevers (caused by substrate under-cutting during fabrication), there is slight difference between the experimentally observed results and the theoretically calculated frequencies of the fundamental mode, which is given by the equation

$f_{R}=\frac{{1.8751}^{2}}{2\pi\sqrt{12}}\frac{t}{L^{2}}\sqrt{\frac{E}{\rho}}$ (S1)

where f_R_ is the fundamental resonance frequency, t and L are thickness and length of the cantilever, E is the Young’s modulus, and ρ is the GaN mass density. In order to include effects of the overhang, a modified equation utilizing the overhang length, ΔL, given as^1^

$f_{R}=\frac{{1.8751}^{2}}{2\pi\sqrt{12}}\frac{t}{{(L+\Delta L)}^{2}}\sqrt{\frac{E}{\rho}}$ (S2)

is used to estimate the microcantilever effective mass. Using the experimentally measured resonance frequency values and the parameters shown in Supplementary Table I., the overhang part of the cantilevers was calculated. Since the length of the cantilever increased by ΔL due to the overhang, effective mass (m) can be calculated using the width of the cantilever (w) as

$m=0.236 (w\times t\times\left( L+\Delta L \right))\times\rho$ (S3)

The Duffing equation is frequently used for describing and estimating nonlinear characteristics of micro and nanoelectromechanical systems with a cubic nonlinearity originating from intrinsic geometric and inertial nonlinearities^2-4^. The equation is given by

$m\ddot{x}+\Gamma\dot{x}+kx+\alpha x^{3}=F\cos\omega t$ (S4)

where *k* is the effective spring constant given as$k=m{\omega_{0}}^{2}=m{(2\pi f_{R})}^{2}$. $\Gamma$ is the linear damping rate given as $\Gamma=\frac{m\omega_{0}}{Q}$, and *m*, *Q* and *ω_0_* are effective mass, quality factor, and fundamental frequency of the resonator, respectively. *F* is the amplitude of external effective force applied by the actuation source, which is either piezoactuator or the lasers utilized in the study. *α* denotes the cubic parameter also known as Duffing constant. Equation 1 is solved using secular perturbation theory, as given by

$x_{0}^{2}=\frac{{(\frac{F}{2k})}^{2}}{{(\frac{\omega-\omega_{0}}{\omega_{0}}-\frac{3\alpha}{8k}x_{0}^{2})}^{2}+{(\frac{1}{2Q})}^{2}}$ (S5)

where $x_{0}$ is the magnitude of the motion. For our GaN microcantilevers, the embedded HFET is utilized to transduce the tip deflections proportionally into electrical signals. Therefore, drain-source voltage (V_DS_) measurements directly correspond to microcantilever oscillation amplitude. Using a deflection sensitivity constant (Tc with unit of m/V), V_DS_ can be added in equation 2 ($x_{0}=V_{DS}T_{C}$). Moreover, in case of piezoactuator based actuation, the amplitude of external force (F) can be replaced with $F = m\omega^{2}V_{Piezo}G_{Piezo}$ where $V_{Piezo}$ and $G_{Piezo}$are the amplitude of the ac bias applied to the piezoactuator and the displacement coefficient of the piezoactuator, respectively. After substituting these parameters into equation 2, the oscillation amplitude can be presented as

${(V_{DS}T_{C})}^{2}=\frac{{(\frac{m\omega^{2}V_{Piezo}G_{Piezo}}{2k})}^{2}}{{(\frac{\omega-\omega_{0}}{\omega_{0}}-\frac{3\alpha}{8k}{(V_{DS}T_{C})}^{2})}^{2}+{(\frac{1}{2Q})}^{2}}$ (S6)

The resonance amplitude of the cantilevers at a certain drive bias reaches its maxima at the frequency of ω_max_, also called drop frequency ω_drop_ which yields^3^,

$\omega_{max}=\omega_{drop}=\omega_{0}+\frac{3\alpha}{8m\omega_{0}}{(V_{DS}^{max}T_{C})}^{2}$ (S7)

where V_DS_^max^ is the maxima of resonance peak in terms of drain-source voltage change. This equation is also known as the backbone curve.

The displacement responsivity of the AlGaN/GaN HFET embedded on the GaN microcantilever strongly depends on the applied gate voltage. Therefore, T_C_, defined as oscillation amplitude per unit magnitude of the ac drain-source voltage V_DS_, is estimated for each experimental resonance curve of the microcantilevers excited with the piezoactuator using the equation^5^

$T_{C}= \frac{F}{V_{DS}^{max}k}Q\approx$ $\frac{V_{Piezo}G_{Piezo}Q}{V_{DS}^{max}}$ (S8)

T_C_ values at different drive amplitudes are calculated using the other parameters V_DS_^max^ , Q, ω_0,_ G_Piezo_, m and ω_drop_ values for the GaN microcantilever with dimensions 250 × 100 μm. Utilizing the strategy discussed in Ref. 5^5^, cubic constant, α, can be determined by fitting the backbone curve equation into the drop frequencies collected from the experimental data of the microcantilever at various excitation biases (V_Piezo_), using the least square fitting approach., The estimated cubic parameter and the modeling results can be seen in Supplementary Table I and Fig S15, respectively.

At drive forces below the critical amplitude, $x_{crit}$, the resonator resonance response stays in linear regime. Increasing the drive force causes a hysteresis response in resonance behavior and two bifurcation points appear in forward and backward frequency sweeps. The critical amplitude defined as the minimum oscillation amplitude at where the bifurcations are distinct, is given by^3^

$x_{crit}=\sqrt{\left| \frac{8}{3\sqrt{3}}\frac{k}{\alpha Q} \right|}$ (S9)

The calculated critical amplitude is calculated as 2.81 µm. The microcantilever critical oscillation amplitude ($x_{crit}$) at the cantilever resonance frequency can be also expressed as

$x_{crit}=\frac{F Q}{k}=\frac{m{\omega_{crit}}^{2}V_{Piezo}G_{Piezo} Q}{k}$ (S10)

where ω_crit_ is the resonance frequency at the critical amplitude, given by^3^

$\omega_{crit}=\omega_{0}-\frac{3\alpha}{8k}{(x_{crit})}^{2}$ (S11)

Using Supplementary equation S11 and S10, the critical piezoactuator voltage is calculated as V_Piezo_Crit_ = 13.53 mV. After estimation the critical piezoactuator bias, we calculated the bistability regime of the microcantilever under study. Only in the bistable regime, the supplementary equation S6 gives three solutions, while it has one solution in the stable regime^3^. Therefore, we computed nonlinear curves by increasing V_Piezo_ by 100 µV from 13.5 mV (the critical piezoactuator bias) to 38 mV (way above the critical voltage). Then, we identified the first and last frequencies of the bistable regime in which three solutions of supplementary equation S6 exist. These frequencies are the forward and backward bistable frequency diagram demonstrated in Fig. 8c of the main manuscript.

**Supplementary Table I.** Summary of the parameters used in calculations

| **Parameter** | **Value** |
| --- | --- |
| GaN density (kg m^-3^) ^6^ | 6.15×10^3^ |
| Piezoactuator displacement coefficient - G_Piezo_ (m/V) | 2.2×10^-8^ |
| Young’s modulus of GaN (Pa) ^6^ | 210×10^9^ |
| Acoustic velocity of GaN (m/s) ^6, 7^ | 8000 |
| 790 nm laser beam radius (m) | 10×10^-6^ |
| Effective mass (kg) | 48.02×10^-12^ |
| Quality factor (after Au deposition) | 9450 |
| Estimated Cubic Parameter (kg m^-2^ s^-2^) | -9.20×10^6^ |


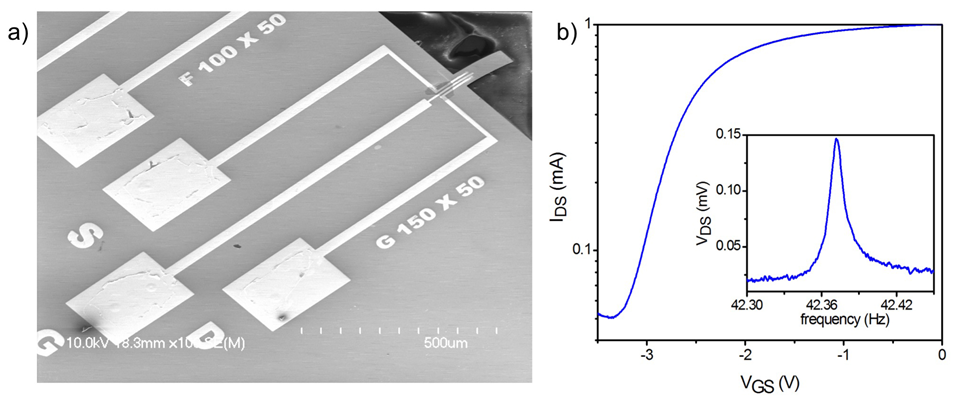


**Figure S1.** a) SEM image of the microcantilever with dimensions of 150 × 50 *µ*m, showing hardening type non-linearity. b) Id-Vg and resonance characteristics (inset) of the cantilever. The resonance frequency and quality factor of the resonator are 42.37 kHz and 4600, respectively.


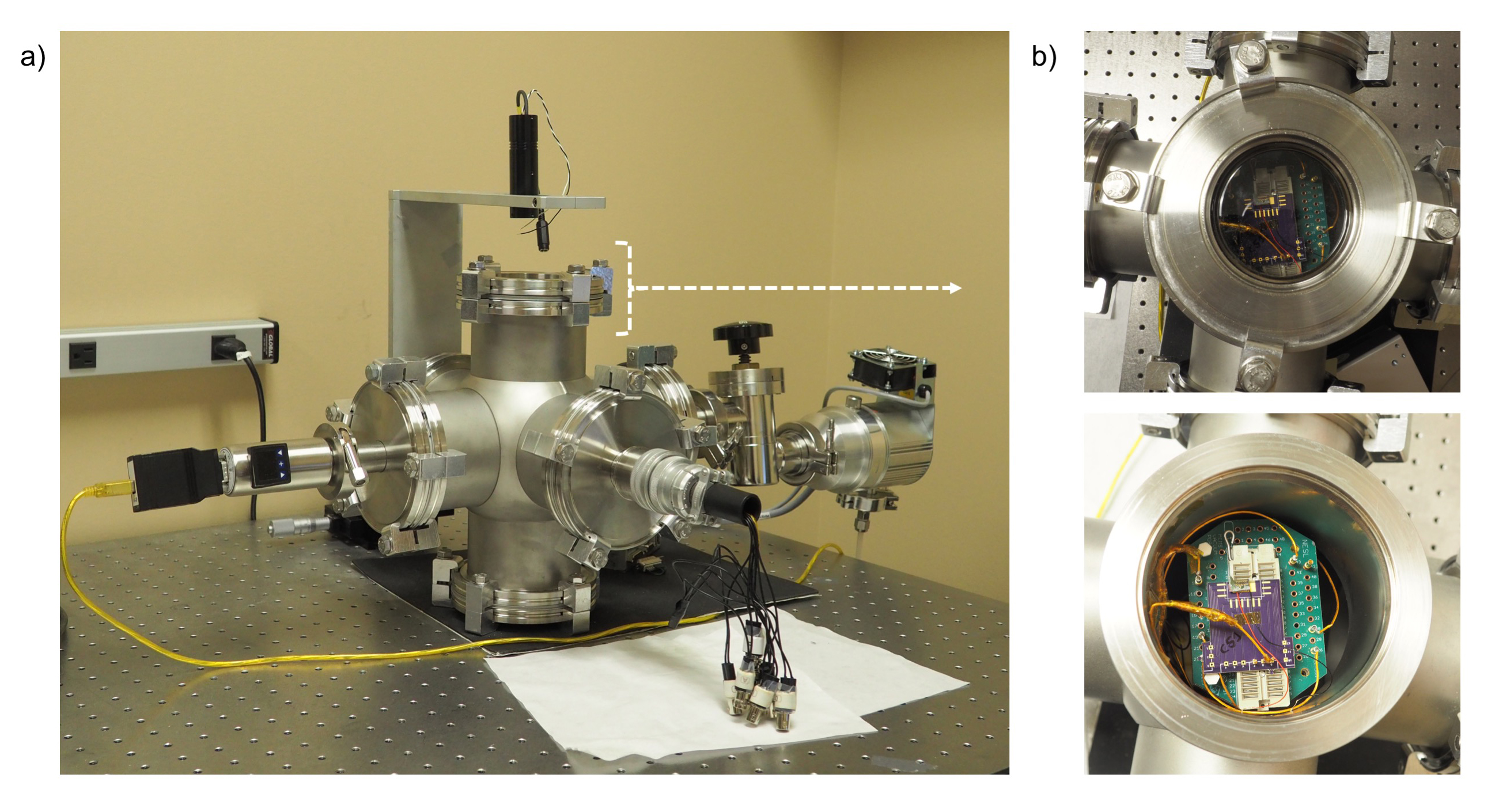


**Figure S2.** a) The vacuum chamber setup utilized in the characterization and mechanical memory experiments. b) Top view images of the highlighted (dotted bracket) part of the vacuum chamber shown in (a). The wire boned microcantilever chip was placed in the home-built vacuum chamber for testing. The top and bottom images display the top view of the vacuum chamber with the view port when it is connected and disconnected, respectively.


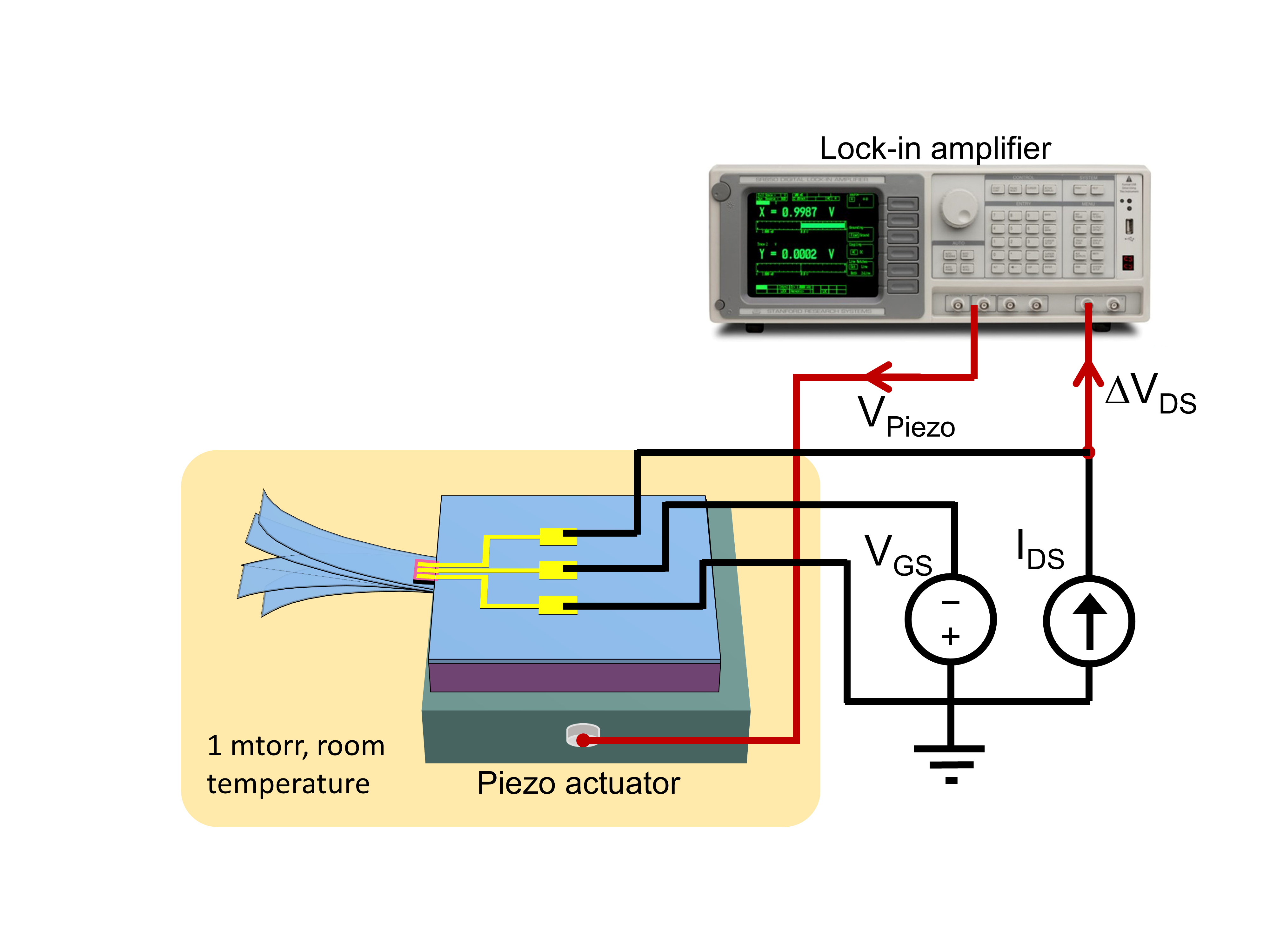


**Figure S3.** Schematic of the experimental setup showing piezo actuator excitation to switch cantilever states.


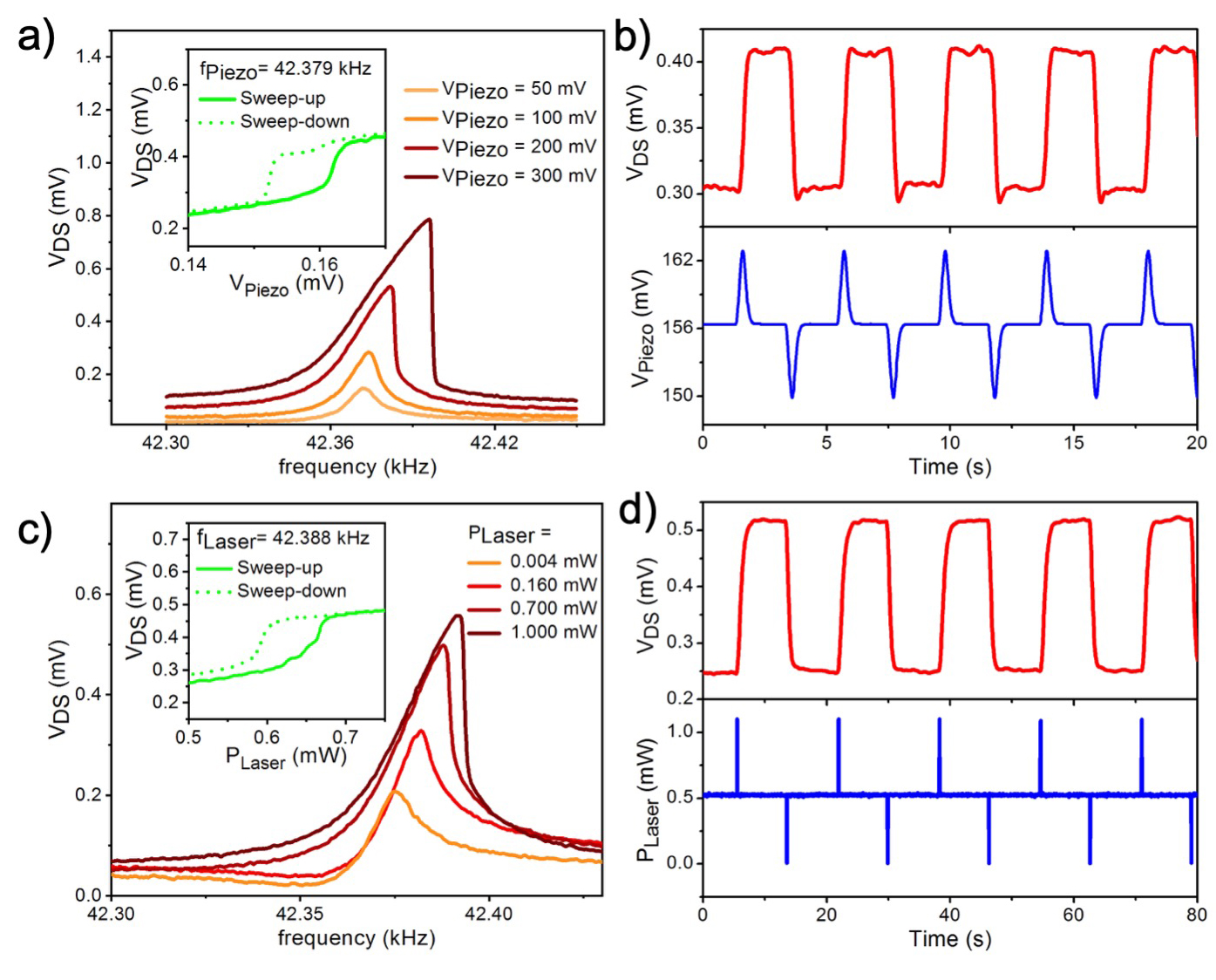


**Figure S4.** a) Resonance curves of GaN microcantilevers with dimensions of 150 × 50 µm, showing hardening type non-linearities, under different piezoactuator biases. Due to the hardening characteristics of the microcantilever, all resonance curves are swept from low frequencies to high frequencies (sweep-up).  Inset shows hysteresis behavior at a fixed frequency of 42.379 kHz. b) Switching the cantilever’s bistable states using piezoactuator biased at 157 mV to keep the cantilever in the bistable region. ±6 mV was added to the signal for 500 ms to switch the cantilever from ON to OFF and vice-versa. c) Hardening type nonlinearities of photoacoustically (520 nm pulsed laser was employed) actuated GaN microcantilever. Inset displays the hysteresis behavior at the frequency of 42.388 kHz. d) Photoacoustic guided switching operations of GaN microcantilevers. The base laser power was 550 mW for bistable region operation, and the laser power was changed by +450 and -500 mW to switch from ON and OFF state and vice-versa.


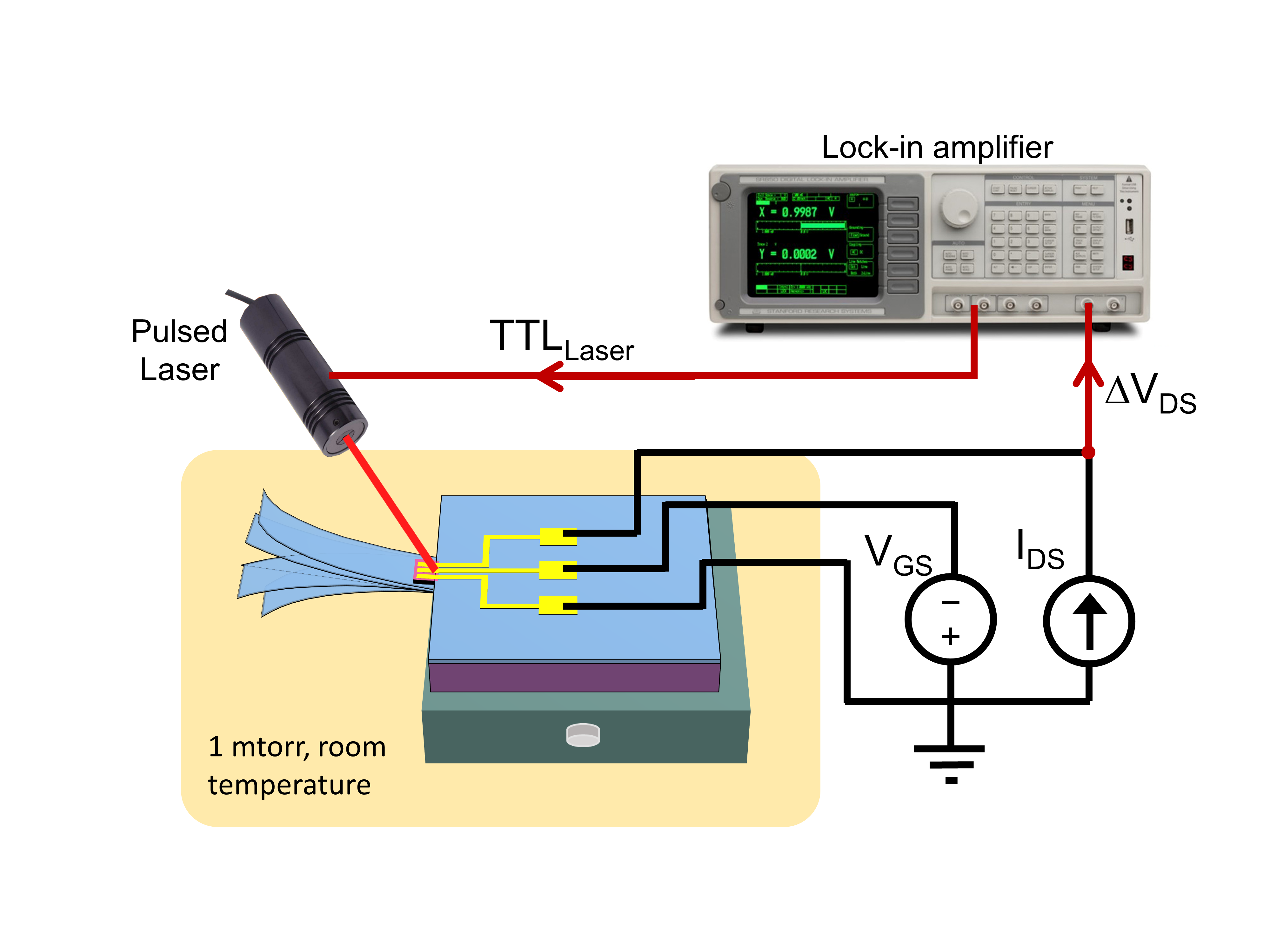


**Figure S5.** Illustration of experimental setup used to excite the microcantilever with a 520 nm pulsed laser.


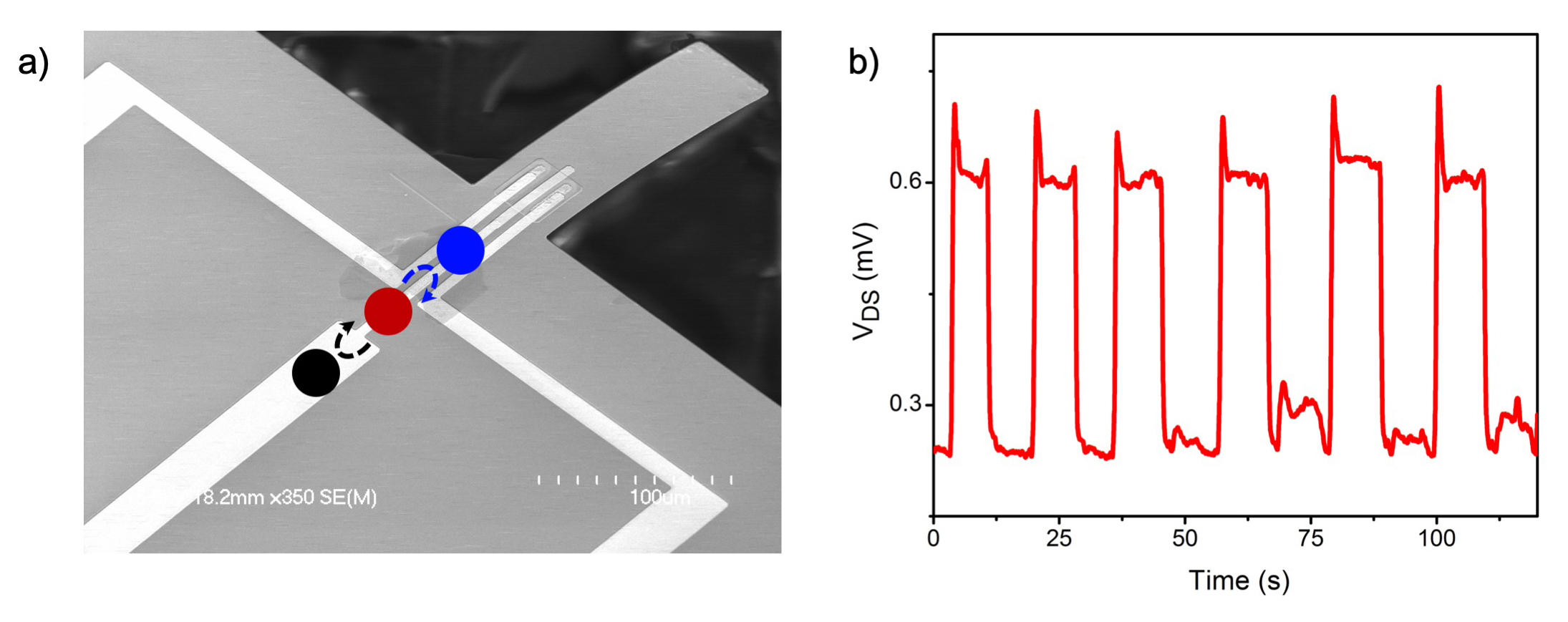


**Figure S6.** Laser location based dynamic switching operations with the microcantilever with dimensions of 150 × 50 µm. a) Illustration of the laser beam positions on the SEM image of the microcantilever. b) Dynamic mechanical memory operations with the hardening type microcantilever repeatedly by manually adjusting the laser locations further and away from the cantilever. To go from low to high state, the laser beam focused on cantilever surface, indicated by red dot in (a), was moved to higher bistable region (blue dot) for 1 second, and brought back the its original position. To switch the cantilever off, same moving procedure to the lower bistable region (marked by black dot) was done as shown black arrow in (a).


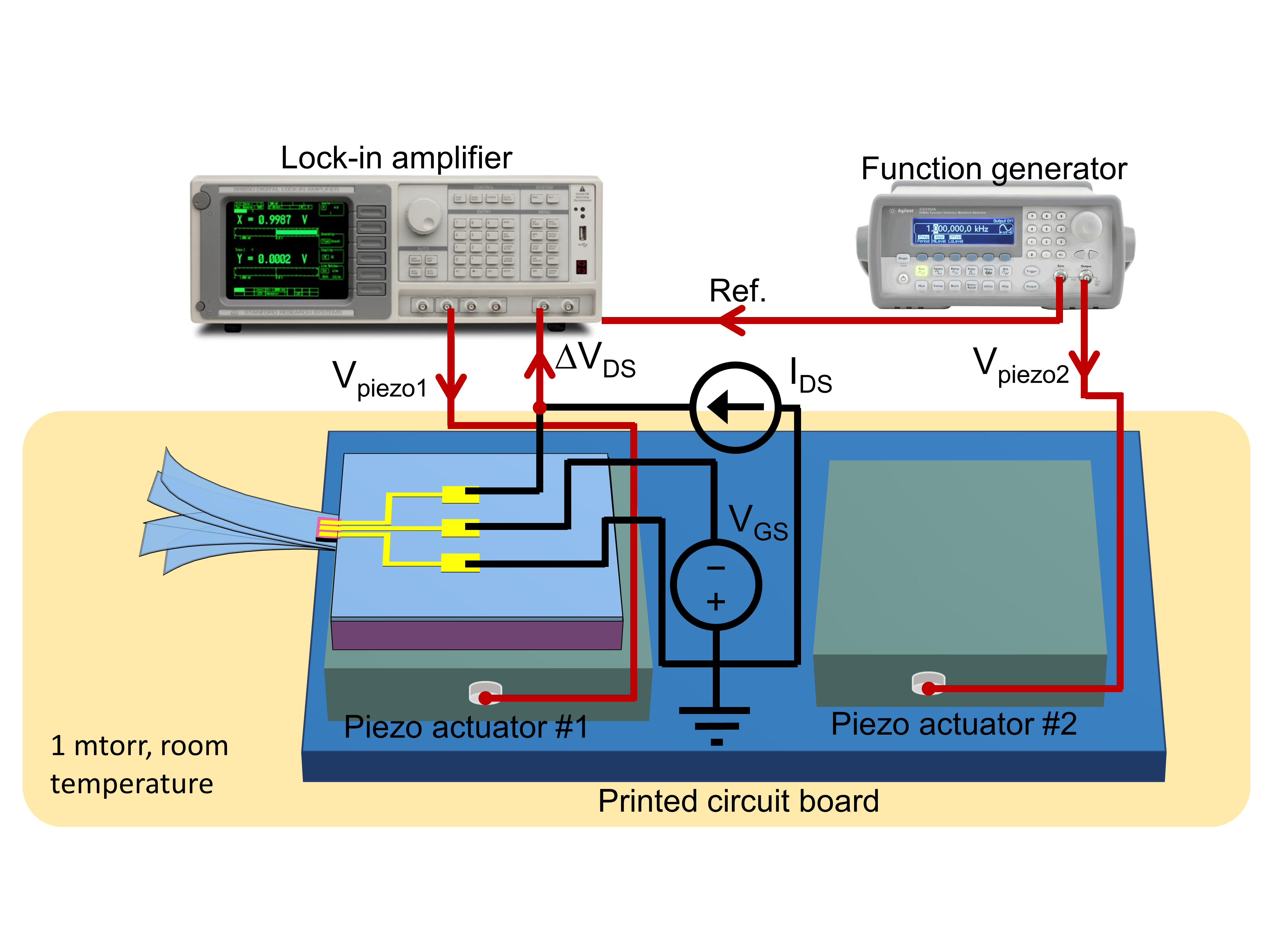


**Figure S7.** Schematic of experimental setup utilized to demonstrate microcantilever switching with two piezo actuator. The piezo actuator #2 is attached to the printed circuit board (PCB) approximately 2 cm away the piezo actuator #1. The biases used for the piezo actuators have same frequency of 15.974 kHz.


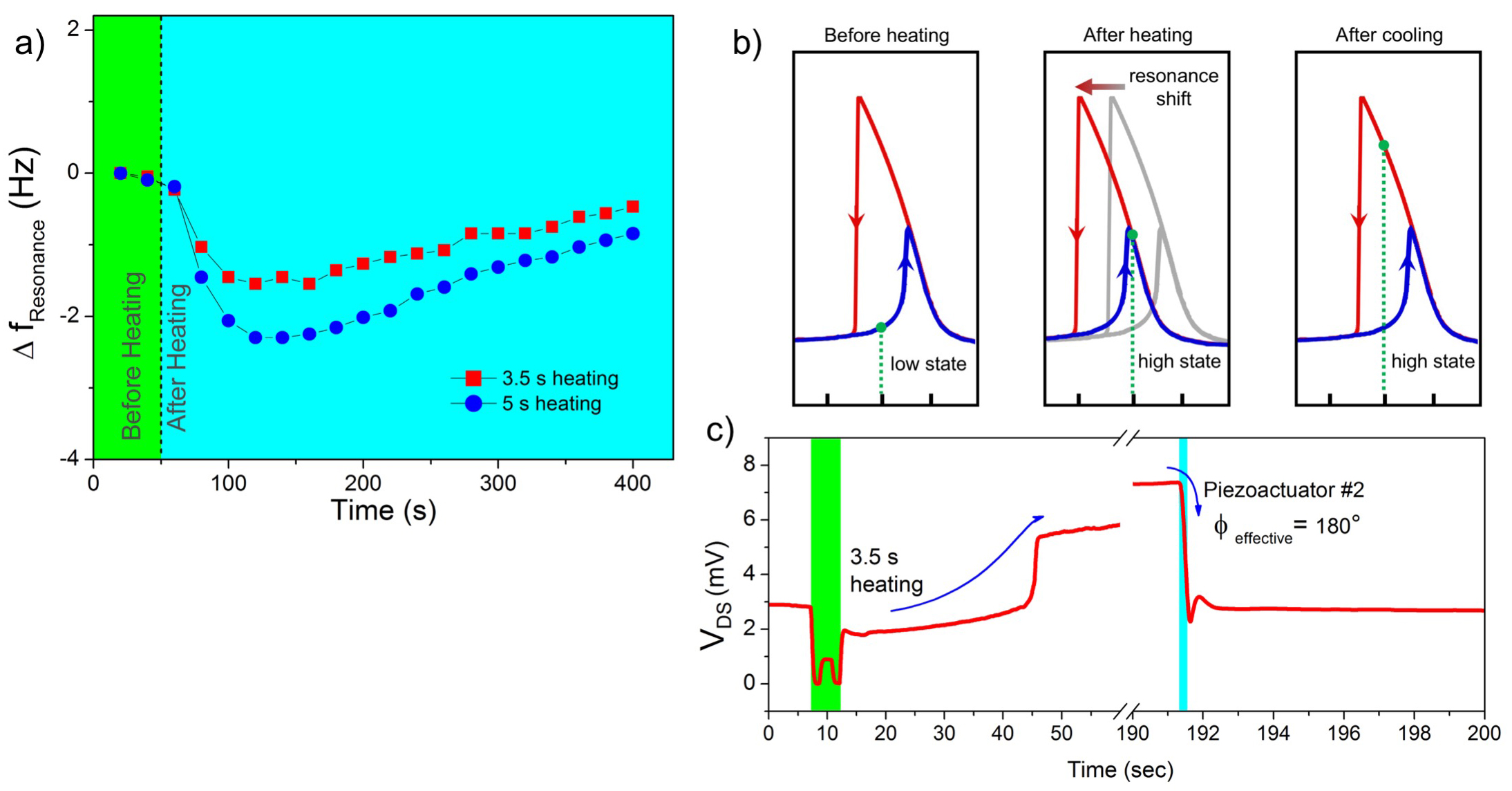


**Figure S8.** Changing the cantilever resonance (excited with a piezoactuator biased at 76 mV) status with a heater. a) Shift in the resonance frequency as a function of time due to heating the microcantilever using a ceramic heater. The microcantilever was heated for 3.5 seconds (red squares) and 5 seconds (blue circles) initially and the variation in frequency were noted with time. b) Graphical explanation of the heating triggered switching. Before heating, the microcantilever is in the low state at the operation frequency in the bistable regime. Upon heating, the bistable operation frequency shift to the stable regime, due to shift in the resonance characteristics. Therefore, the oscillation amplitude increases as shown as by the green dot. When the cantilever now cools down, shifting the resonance curve to its original position, the amplitude remains in the high state of the bistable region. c) Experimental demonstration of switching operations using a ceramic heater turned on for 3.5 seconds (shown as the green area) to switch the cantilever on (happened at ~45 s). The first piezoactuator (15.975 kHz, 76 mV) was used to maintain the microcantilever oscillations in the bistable region. Destructive interference form a second piezoactuator biased at 500 mV and 180° phase was used to turn the cantilever off (happened ~ 191.5 s).


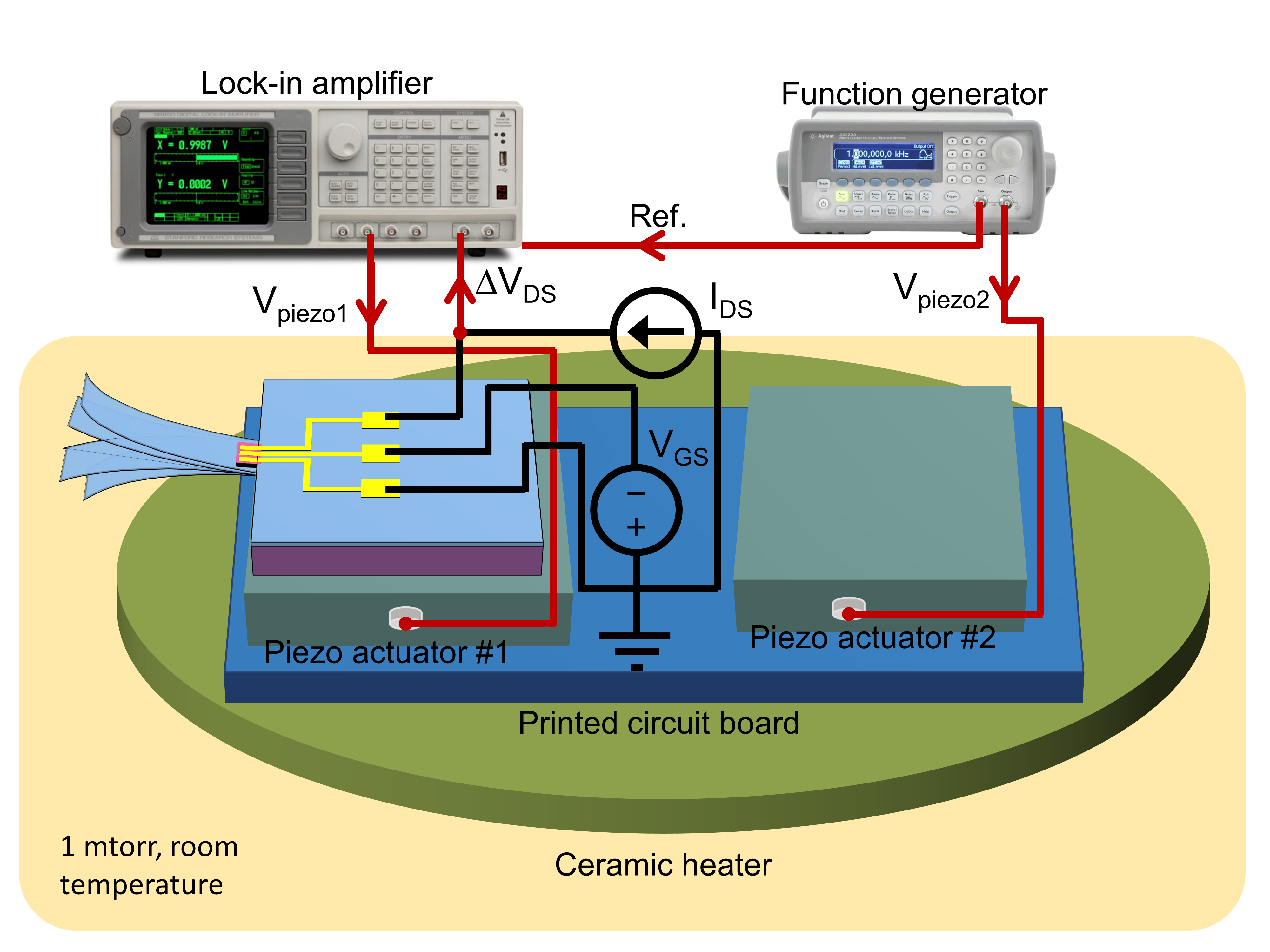


**Figure S9.** Schematic of experimental setup utilized to demonstrate microcantilever switching with two piezo actuator and a ceramic heater. The ceramic heater was turn on for 3.5 seconds to heat the cantilever up, which results in switching the cantilever from off state to on state.


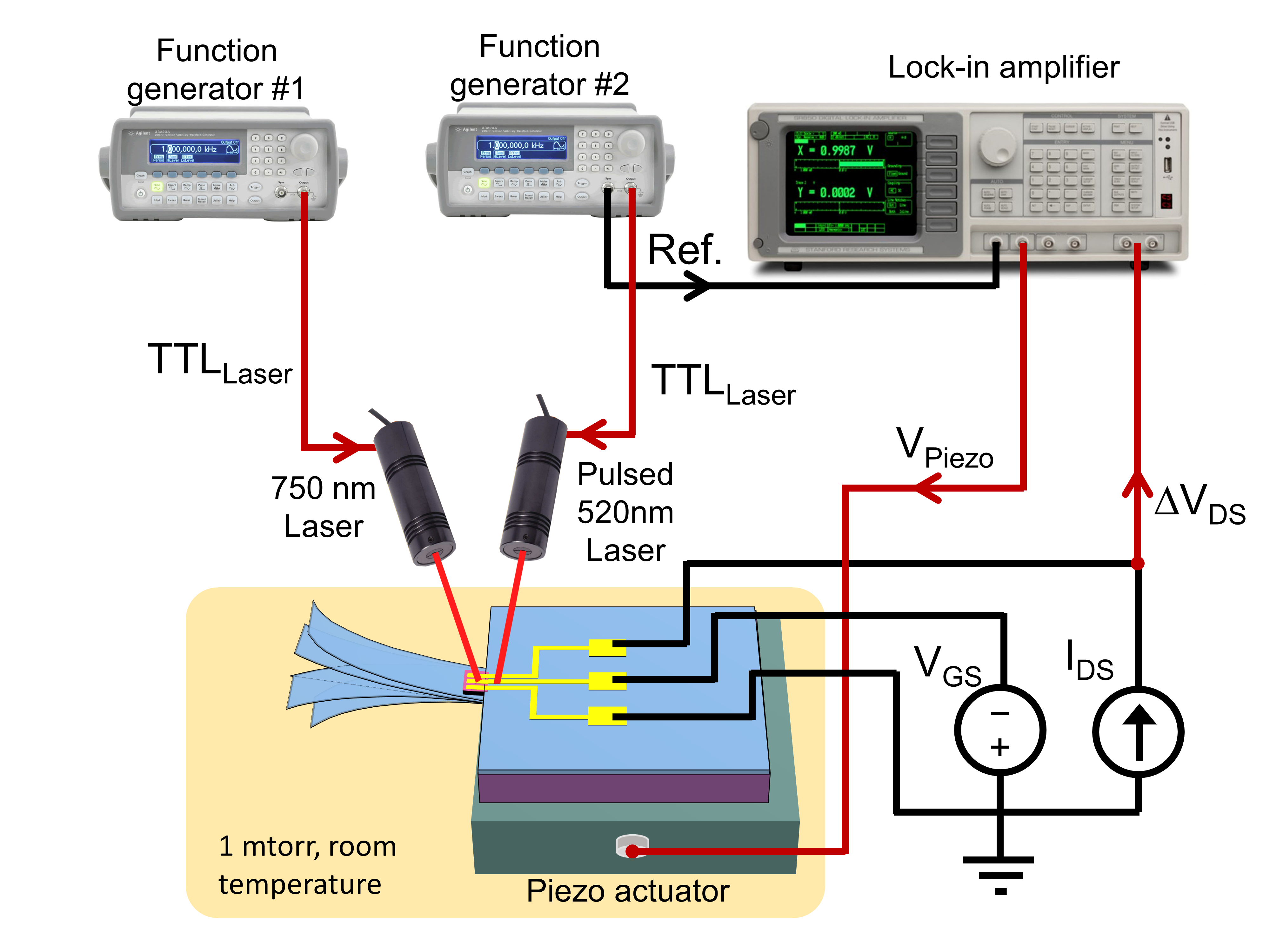


**Figure S10.** Experimental schematic of heating and destructive interference assisted cantilever switching with 2 lasers. A low power (~50 μW) laser with 790 nm wavelength was focused on the microcantilever HFET, while the 520-nm laser (600 μW) was carefully focused ~100 μm away from the cantilever base to ensure that the thermal effects caused by it are negligible. To change the cantilever current state from low to high, the 790-nm laser (steady, no pulsing) was kept on for 300 ms using the function generator #1 to heat the cantilever. The 520-nm laser with a pulsed frequency of 15.684 kHz and 180° phase shift relative to the piezo actuator signal was kept on for 50 ms (using the function generator #2) to make the cantilever switch from high state to low state by destructive interference


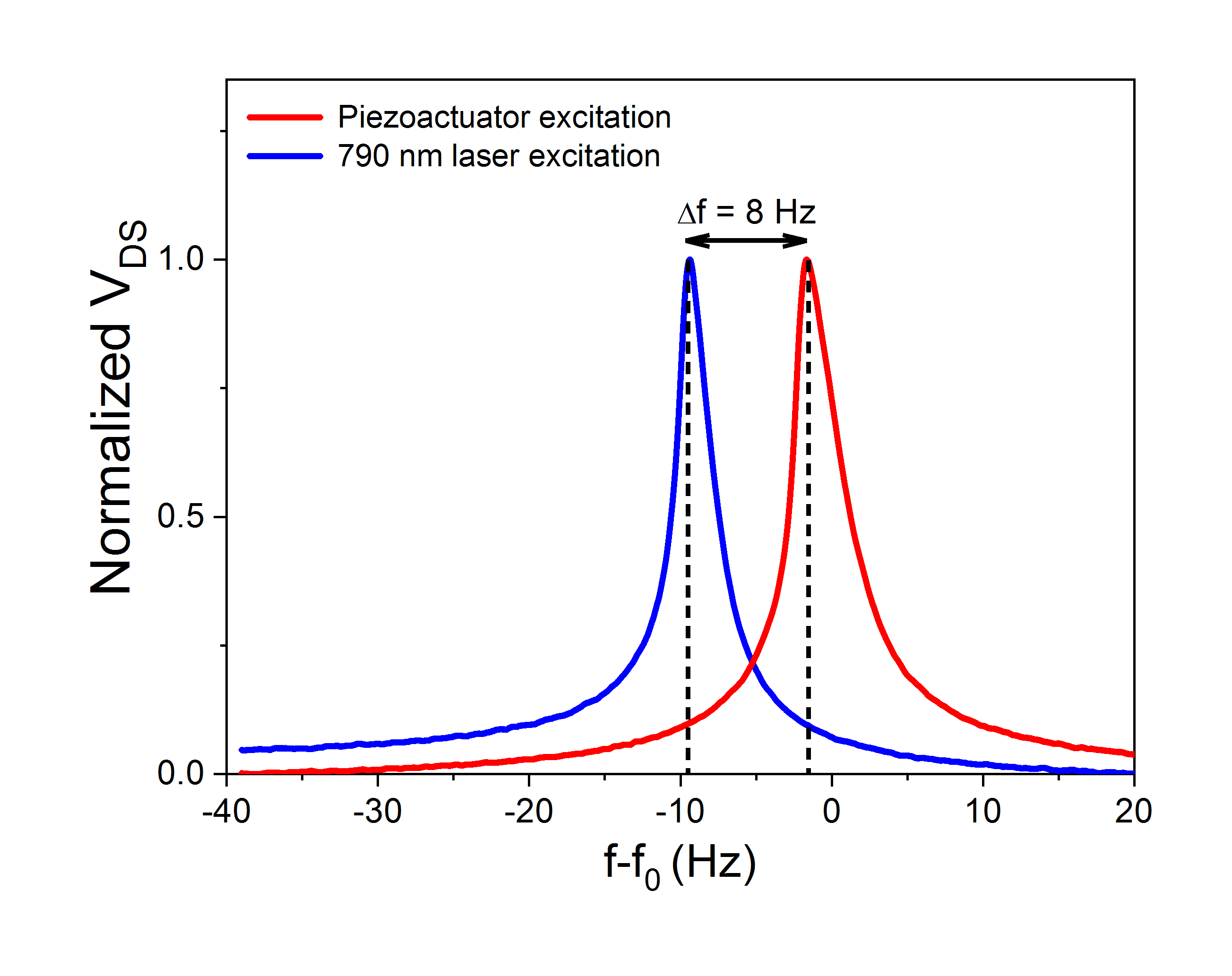


**Figure S11.** Shift in the resonance frequency of the microcantilever with dimensions of 250 × 100 μm, due to temperature rise caused by low power 790 nm laser incidence. It should be noted that photocurrent generated at the cantilever HFET under 790 nm laser exposure does not alter the resonant behavior since the normalized off-resonance noises with piezo actuator and the 790 nm laser are approximately same.


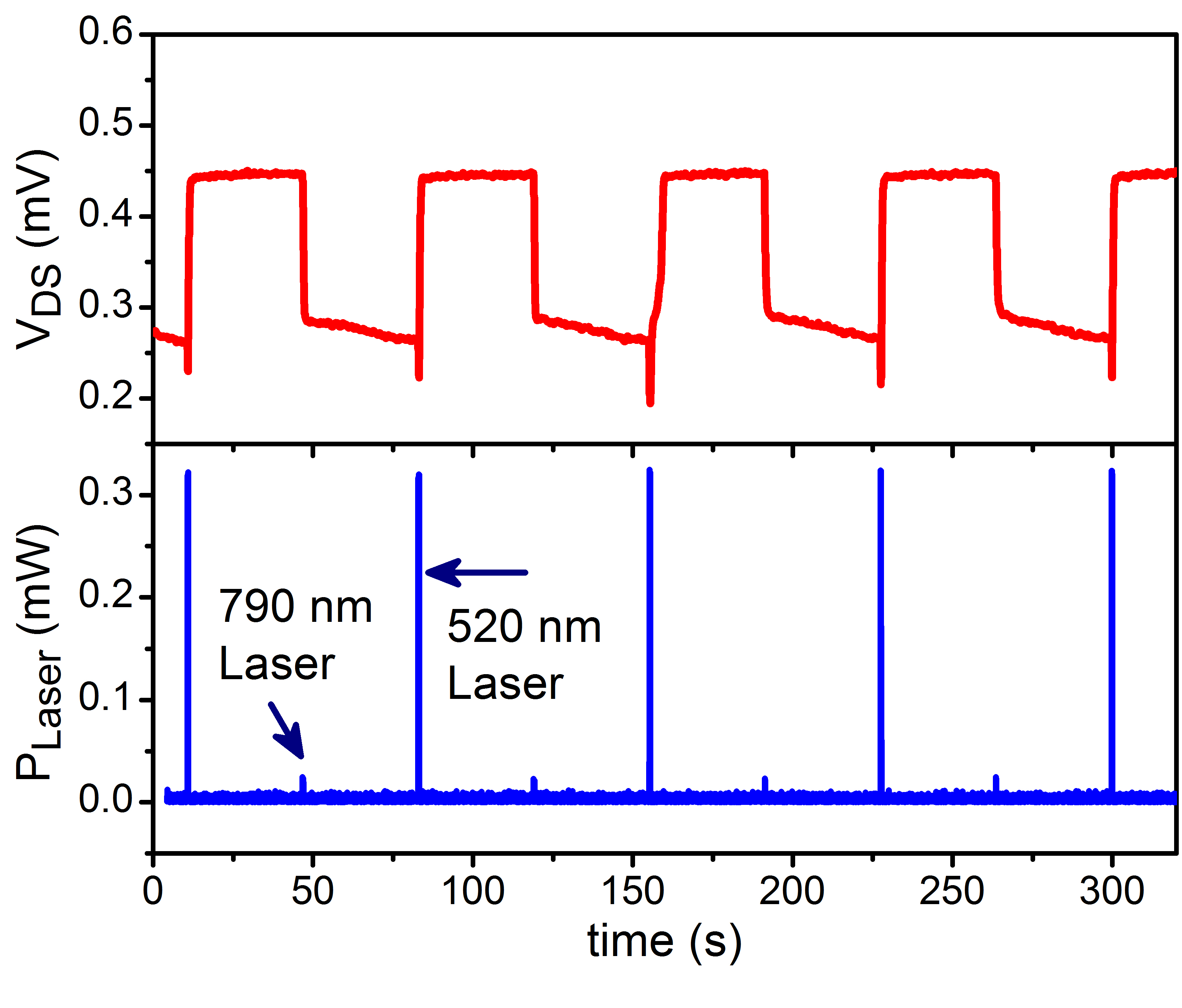


**Figure S12.**  ON-OFF switching operations in a microcantilever (hardening type) with dimensions 150 × 50 μm using two lasers, while the piezo actuator was used to provide primary excitation o the cantilever for bistable region operation. The 520 nm laser was turned on for 250 ms to switch the cantilever ON, while the 790 nm laser was pulsed at the frequency of 42.360 kHz (with a phase of 180°) and pulse-width of 250 ms to turn the cantilever OFF. The switch-on operation is solely driven by heating effects, while destructive interference was utilized for switch-off operation.


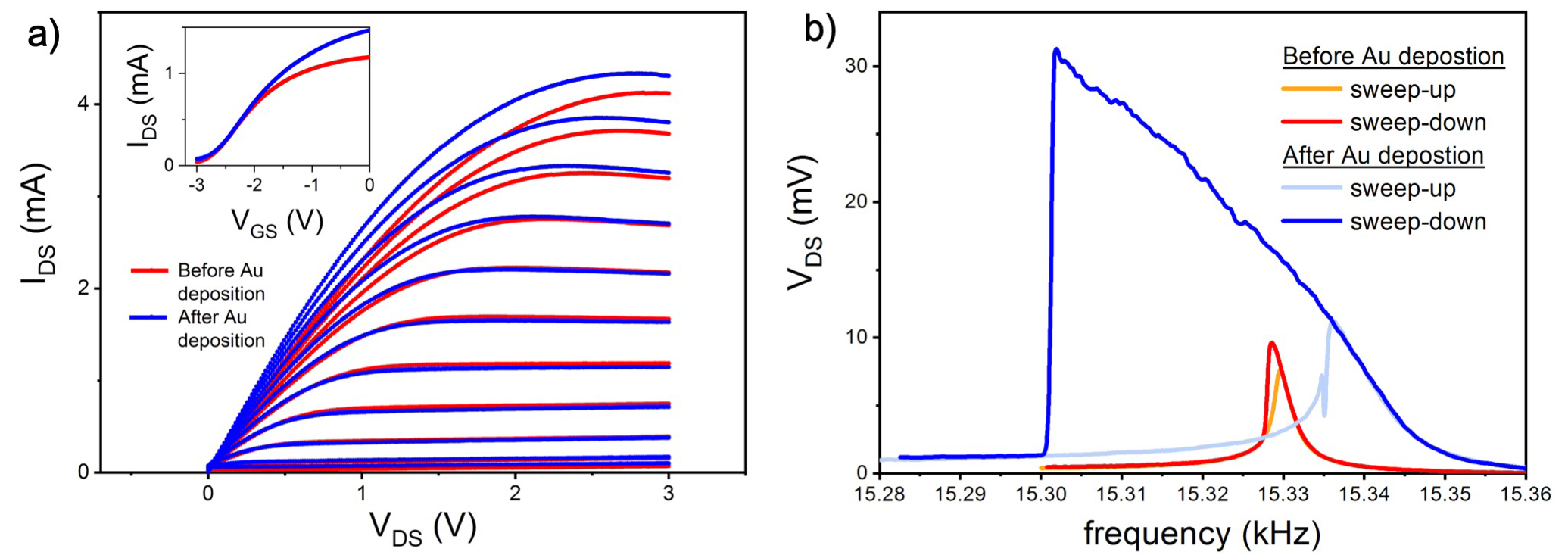


**Figure S13.** Enhancing photoacoustic signal with Au plasmonics a) I-V characteristics of the AlGaN/GaN HFET transducer at the base of the microcantilever with dimensions of 250 × 100 μm before (red) and after (blue) Au nanoparticle deposition. Inset shows I_DS_-V_GS_ behavior of the HFET. There is a slight increment in the saturation drain current due to increased conduction caused by the Au deposition. b) Resonance characteristics of the microcantilever excited using the 790 nm laser with 45 µW μm before and after Au nanoparticle deposition. The laser beam was focused very close to the cantilever HFET in the measurements. Sweep-up (low to high) and sweep-down (high to low) resonance response of the microcantilever is shown as orange and red solid curves before depositing Au nanoparticle, respectively. Due to strong plasmonic photoacoustic signal amplification, the cantilever resonance response was enhanced after Au deposition, as shown in sweep-up (light blue) and sweep-down (blue) curves.


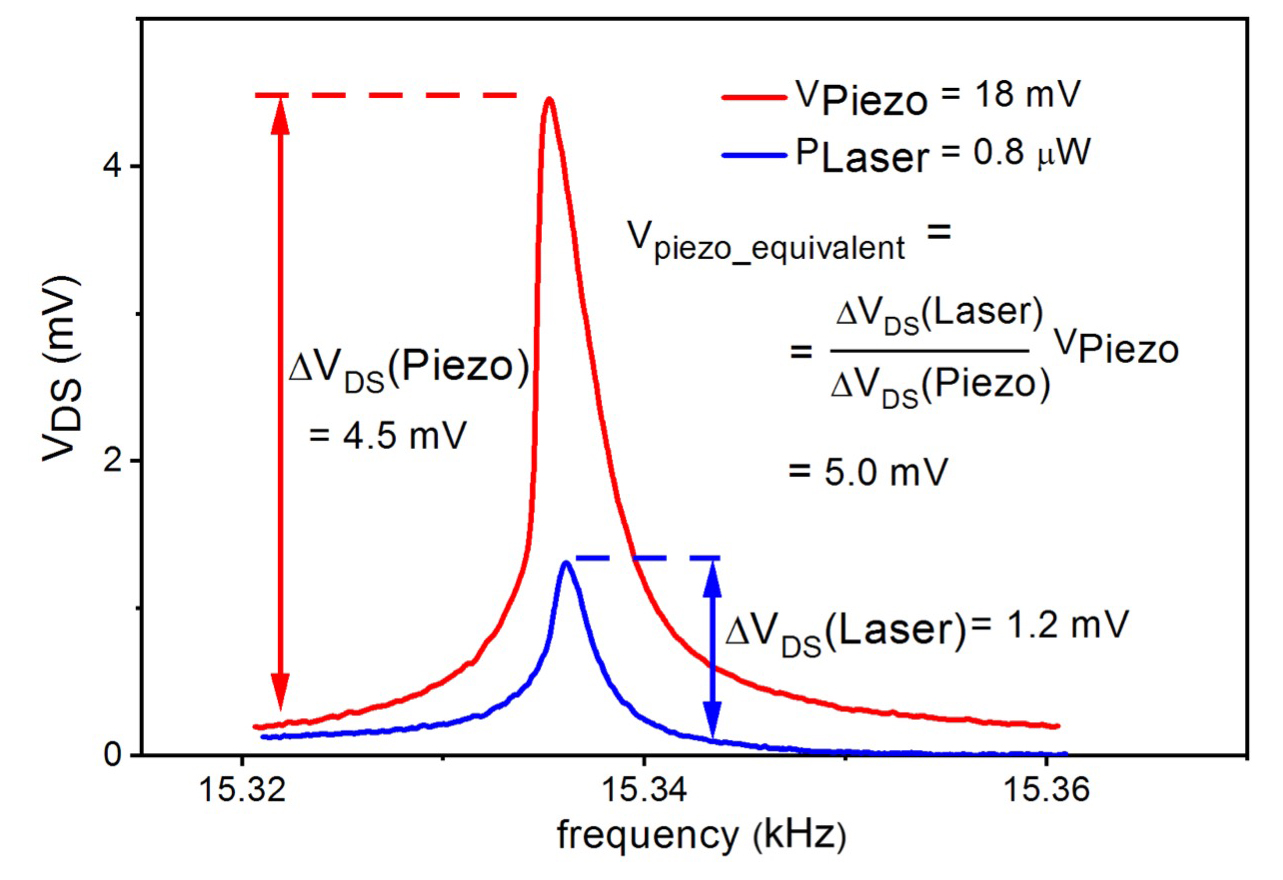


**Figure S14.**   Direct comparison of the resonance curves obtained using laser based (blue) and piezoactuator (red) excitations, to find out conversion factor between the laser power and the piezoactuator bias voltage.  The 790 nm laser with 800 nW power was used for photoacoustic excitation. From the calculation in the inset, it is found that the equivalent resonance amplitude caused by 800 nW laser based photoacoustic excitation would be caused by a piezoactuator bias V_Piezo_ = 5.0 mV.


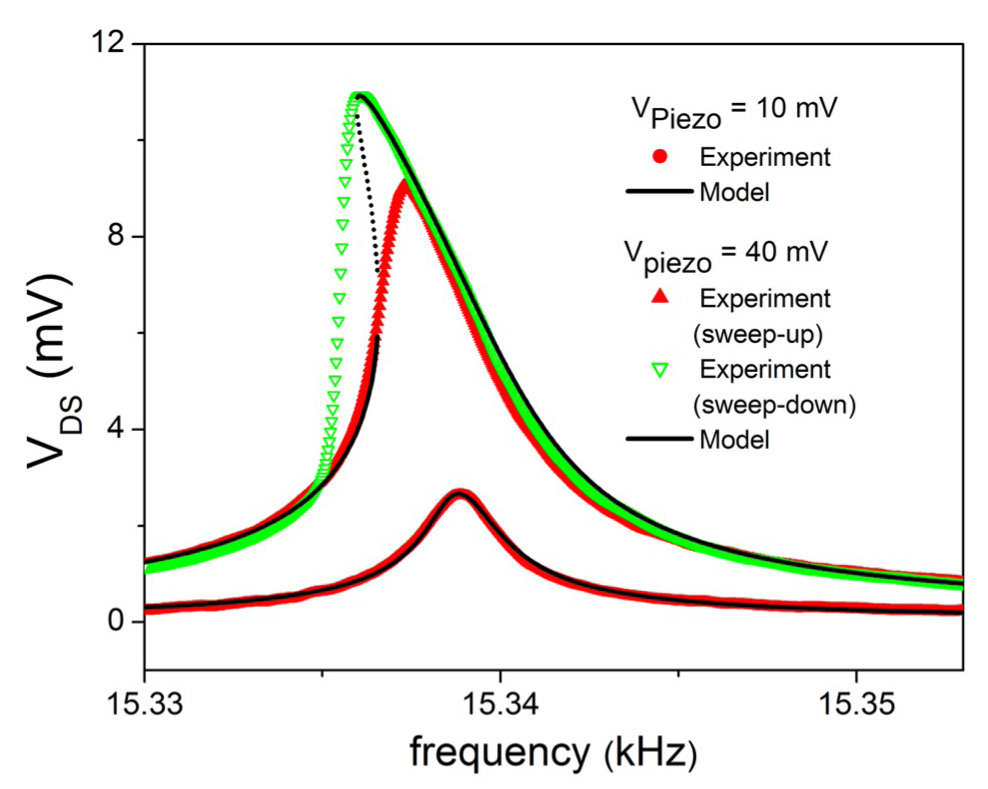


**Figure S15.** Theoretical fits and experimental resonance curves of the GaN microcantilever with dimensions of 250 × 100 μm, in the linear and non-linear regimes. Linear resonance characteristics of the cantilever excited with V_Piezo_ = 10 mV as red circles. Sweep-up (low to high) and sweep-down (high to low) hysteric resonance response of the microcantilever in bistable regime due to higher excitation bias (V_Piezo_ = 40mV) are shown as filled-red and empty-green triangles. The solid and dotted black lines correspond to the stable and unstable portions of the theoretical fit, respectively. The values of the deflection sensitivity constant (Tc) used in the presented model curves are 7.1×10^-4^ m/V and 6.8×10^-4^ m/V for the piezoactuator biases of V_Piezo_ = 10 mV and V_Piezo_ = 40 mV, respectively.

**Supplementary Table II.** Specifications of State of the Art MEMS/NEMS Resonator based Memory and Logic Operations

| **#Ref** | **Resonator Structure** | | **M/NEMS Dimensions (μ)** | | | **Operation Conditions (Pressure and Temperature)** | **Resonator Operation Characteristics (Governed Equation)** | **Resonance Frequency** | **Detection**  **Method** | **Excitation**  **Method** | **Switching Power/Energy (W/J)** | **Resonator Mass (kg)** | **Switching Energy per Unit Mass (****J/kg)** | **Operation Speed (s)**  **(Experimental and Theoretical)** |
| --- | --- | --- | --- | --- | --- | --- | --- | --- | --- | --- | --- | --- | --- | --- |
|  |  |  | **Length** | **Width** | **Thickness** |  |  |  |  |  |  |  |  |  |
| ^8^ | | DCB* | 8 | 0.3 | 0.2 | 0.275 K | Softening (Duffing) | 23.568 MHz | Electromotive | Magnetomotive | - | - | - | - |
| ^9^ | | DCB | 15 | 0.3 | 0.5 | 1 mTorr | Softening (Duffing) | 4.83 MHz | Electrostatic | Electrostatic | - | - | - | ~1 ms (experimental)  ~20 µs (theoretical) |
| ^10^ | | DCB | 5 | 0.5 | 0.11 | 10 mTorr | Hardening  (Duffing) | 10.075 MHz | Optical | Electrostatic | - | - | - | ~0.2 ms (experimental)  ~40 µs (theoretical) |
| ^11^ | | DCB | 20 | 0.3 | 0.5 | 10 mTorr | Hardening | 3.145 MHz | Electrostatic | Electrostatic | ~10^-17^ J | ~10^-14^ | ~10^-3^ | ~0.2 ms (experimental) |
| ^12^ | | DCB | 500 | 3 | 30 | 1 Torr  RT* | Linear Region | 117 kHz | Electrostatic | Electrostatic & Electrothermal | ~10^-2^ W | - | - | ~4 ms (theoretical) |
| ^13^ | | DCB | 20 | 0.3 | 0.5 | - | Linear Region | 3 MHz | Electrostatic | Electrostatic | ~10^-17^ J | ~10^-14^ | ~10^-3^ | ~0.2 µs (theoretical) |
| ^14^ | | DCB | 500 | 3 | 30 | 200 mTorr | Softening | 124 kHz | Electrostatic | Electrostatic | ~10^-13^ J | ~10^-10^ | ~10^-3^ | ~60 ms (theoretical) |
| ^15^ | | DBC | 500 | 3 | 30 | < 1 Torr | Softening | 121.5 kHz | Electrostatic | Electrostatic & Electrothermal | ~10^-5^ W | - | - | ~50 ms (theoretical) |
| ^16^ | | DCB | 15 | 0.75 | 1.85 | 40 µTorr | Linear Region | 23.35 MHz | Electrostatic | Electrostatic & Electrothermal | ~10^-4^ W | - | - | ~4.8 ms (experimental)  ~25 µs (theoretical) |
| ^17^ | | DCB | 260 | 84 | 1.35 | 150 nTorr  2.5 K | Hardening  (Parametric)* | 137 kHz | Piezoelectric | Piezoelectric | ~10^-11^ W | ~10^-10^ | ~10^-1^ | ~4 s (experimental) |
| ^18^ | | DCB | 260 | 84 | 1.35 | 0.75 µTorr  2 K | Hardening  (Parametric) | 181 kHz | Piezoelectric | Piezoelectric | - | - | - | - |
| ^19^ | | DCB-OC* | 10 | 0.5 | 0.11 | 100 µTorr | Softening  (Duffing) | 8 MHz | Optical | Optical Cavity based waveguides | ~10^-6^ J | - | - | - |
| ^20^ | | DCB  NC* | 13  6.5 | 0.35  0.35 | 0.28  0.28 | 2 mTorr | Hardening  Softening | 17.7 MHz  11.1 MHz | Capacitive | Electrostatic | - | - | - | - |
| ^21^ | | NC | 10 | 0.7 | 0.75 | 80 µTorr | Softening | 7.464 MHz | Electrostatic | Electrostatic | ~10^-15^ J | ~10^-14^ | ~10^-1^ | 200 µs (theoretical) |
| ^22^ | | CD* | 175 | 575 | 25 | 75 mTorr  RT | Hardening  (Duffing) | 8.66 kHz | Capacitive | Electrostatic | - | - | - | - |
| ^23^ | | MC* | 40 | 8 | 0.2 | 75 µTorr | Hardening  (Duffing) | 94.35 kHz | Optical | Piezoactuator | - | - | - | - |
| ^24^ | | MC | 950 | 200 | 5 | 25 µTorr | Hardening  (Duffing) | 3.835 kHz | Current Measurements | Magnetic Field | - | - | - | 1 s |
| ^25^ | | MC | 1000 | 200 | 5 | 262 µTorr | Hardening | 3.55 kHz | Piezoelectric | Piezo/Magnetic field | - | - | - | - |
| **This work** | | **MC** | **250** | **100** | **1.3** | **1 mTorr**  **RT** | **Softening**  **(Duffing)** | **15.69 kHz** | **Piezotransistive** | **Combined Piezoactuator and Photoacoustic** | **~10^-13^ J** | **~10^-10^** | **~10^-3^** | **~600 ms (theoretical)** |
|  |  | **MC** | **150** | **50** | **1.3** |  | **Hardening (Duffing)** | **42.37 kHz** |  |  | **-** | **-** | **-** | **~100 ms (theoretical)** |

DCB*: Doubly Clamped Beam

MC*: Microcantilever

NC*: Nanocantilever

OC*: Optical Cavity

CD*: Comb Drive

Parametric*: Govern by Mathiu eq.

RT*=Room temperature

**References**

1. Gavan, K. B., Van der Drift, E., Venstra, W., Zuiddam, M. & Van der Zant, H. Effect of undercut on the resonant behaviour of silicon nitride cantilevers. *J Micromech Microengineering* **19**, 035003 (2009).

2. Nayfeh, A. H. & Mook, D. T. in *Nonlinear oscillations* (John Wiley & Sons, 2008).

3. Lifshitz, R. & Cross, M. Nonlinear dynamics of nanomechanical and micromechanical resonators. *Review of nonlinear dynamics and complexity* **1**, 1-52 (2008).

4. Villanueva, L. *et al*. Nonlinearity in nanomechanical cantilevers. *Physical Review B* **87**, 024304 (2013).

5. Bayram, F., Gajula, D., Khan, D., Gorman, S. & Koley, G. Nonlinearity in piezotransistive GaN microcantilevers. *J Micromech Microengineering* **29**, 125011 (2019).

6. Levinshtein, M. E., Rumyantsev, S. L. & Shur, M. S. in *Properties of Advanced Semiconductor Materials: GaN, AIN, InN, BN, SiC, SiGe* (John Wiley & Sons, 2001).

7. Rais-Zadeh, M. *et al*. Gallium nitride as an electromechanical material. *J Microelectromech Syst* **23**, 1252-1271 (2014).

8. Badzey, R. L., Zolfagharkhani, G., Gaidarzhy, A. & Mohanty, P. A controllable nanomechanical memory element. *Appl. Phys. Lett.* **85**, 3587-3589 (2004).

9. Guerra, D. N., Imboden, M. & Mohanty, P. Electrostatically actuated silicon-based nanomechanical switch at room temperature. *Appl. Phys. Lett.* **93**, 033515 (2008).

10. Noh, H., Shim, S., Jung, M., Khim, Z. G. & Kim, J. A mechanical memory with a dc modulation of nonlinear resonance. *Appl. Phys. Lett.* **97**, 033116 (2010).

11. Guerra, D. N. *et al*. A noise-assisted reprogrammable nanomechanical logic gate. *Nano letters* **10**, 1168-1171 (2010).

12. Hafiz, M. A. A., Kosuru, L. & Younis, M. I. Microelectromechanical reprogrammable logic device. *Nature communications* **7**, 1-9 (2016).

13. Wenzler, J., Dunn, T., Toffoli, T. & Mohanty, P. A nanomechanical Fredkin gate. *Nano letters* **14**, 89-93 (2014).

14. Hafiz, M. A. A., Kosuru, L., Ramini, A., Chappanda, K. N. & Younis, M. I. In-plane MEMS shallow arch beam for mechanical memory. *Micromachines* **7**, 191 (2016).

15. Al Hafiz, M. A., Kosuru, L. & Younis, M. I. Electrothermal frequency modulated resonator for mechanical memory. *J Microelectromech Syst* **25**, 877-883 (2016).

16. Kazmi, S. N. *et al*. Tunable nanoelectromechanical resonator for logic computations. *Nanoscale* **9**, 3449-3457 (2017).

17. Mahboob, I. & Yamaguchi, H. Bit storage and bit flip operations in an electromechanical oscillator. *Nature nanotechnology* **3**, 275-279 (2008).

18. Mahboob, I., Mounaix, M., Nishiguchi, K., Fujiwara, A. & Yamaguchi, H. A multimode electromechanical parametric resonator array. *Scientific reports* **4**, 4448 (2014).

19. Bagheri, M., Poot, M., Li, M., Pernice, W. P. & Tang, H. X. Dynamic manipulation of nanomechanical resonators in the high-amplitude regime and non-volatile mechanical memory operation. *Nature nanotechnology* **6**, 726-732 (2011).

20. Uranga, A. *et al*. Exploitation of non-linearities in CMOS-NEMS electrostatic resonators for mechanical memories. *Sensors and Actuators A: Physical* **197**, 88-95 (2013).

21. Chappanda, K. *et al*. A single nano cantilever as a reprogrammable universal logic gate. *J Micromech Microengineering* **27**, 045007 (2017).

22. Yao, A. & Hikihara, T. Logic-memory device of a mechanical resonator. *Appl. Phys. Lett.* **105**, 123104 (2014).

23. Venstra, W. J., Westra, H. J. & van der Zant, Herre SJ. Mechanical stiffening, bistability, and bit operations in a microcantilever. *Appl. Phys. Lett.* **97**, 193107 (2010).

24. Onuta, T., Wang, Y., Long, C. J., Lofland, S. E. & Takeuchi, I. Dynamic state switching in nonlinear multiferroic cantilevers. *Appl. Phys. Lett.* **101**, 043506 (2012).

25. Onuta, T., Wang, Y., Lofland, S. E. & Takeuchi, I. Multiferroic operation of dynamic memory based on heterostructured cantilevers. *Adv Mater* **27**, 202-206 (2015).
